# Targeting Tumor Heterogeneity by Breaking a Stem Cell and Epithelial Niche Interaction Loop

**DOI:** 10.1002/advs.202307452

**Published:** 2024-05-06

**Authors:** Rongze Ma, Deyi Feng, Jing Chen, Jiecan Zhou, Kun Xia, Xiangyin Kong, Guohong Hu, Pengfei Lu

**Affiliations:** ^1^ MOE Key Lab of Rare Pediatric Diseases & Hunan Key Laboratory of Medical Genetics of the School of Life Sciences Hengyang, Hunan 421001 China; ^2^ Institute of Cell Biology University of South China Hengyang, Hunan 421001 China; ^3^ Institute for Future Sciences Hengyang Medical School University of South China Hengyang, Hunan 421001 China; ^4^ School of Life Science and Technology ShanghaiTech University 393 Middle Huaxia Road Shanghai 201210 China; ^5^ The First Affiliated Hospital Pharmacy Department Hengyang Medical School University of South China Hengyang, Hunan 421001 China; ^6^ CAS Key Laboratory of Tissue Microenvironment and Tumor Shanghai Institute of Nutrition and Health University of Chinese Academy of Sciences Chinese Academy of Sciences Shanghai 200031 China

**Keywords:** BMP signaling, drug resistance, FGF signaling, individualized medicine, intra‐tumoral heterogeneity, microenvironment, precision medicine, targeted therapy

## Abstract

Tumor heterogeneity, the presence of multiple distinct subpopulations of cancer cells between patients or among the same tumors, poses a major challenge to current targeted therapies. The way these different subpopulations interact among themselves and the stromal niche environment, and how such interactions affect cancer stem cell behavior has remained largely unknown. Here, it is shown that an FGF‐BMP7‐INHBA signaling positive feedback loop integrates interactions among different cell populations, including mammary gland stem cells, luminal epithelial and stromal fibroblast niche components not only in organ regeneration but also, with certain modifications, in cancer progression. The reciprocal dependence of basal stem cells and luminal epithelium is based on basal‐derived BMP7 and luminal‐derived INHBA, which promote their respective expansion, and is regulated by stromal‐epithelial FGF signaling. Targeting this interaction loop, for example, by reducing the function of one or more of its components, inhibits organ regeneration and breast cancer progression. The results have profound implications for overcoming drug resistance because of tumor heterogeneity in future targeted therapies.

## Graphical abstract

Ma et al. demonstrate that a fibroblast growth factor‐bone morphogenetic protein (FGF‐BMP) signaling positive feedback loop, involving mammary stem cells, luminal epithelial, and niche components, hinders organ regeneration and breast cancer progression, suggesting a new strategy to overcome cancer heterogeneity and drug resistance.

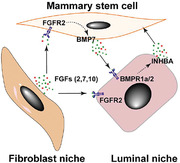

## 1 Introduction

Drug resistance is one of the most significant challenges facing cancer‐targeted therapies.^[^ ^1^, ^2^ ^]^ Although genomic mutations had long been considered as the driving force of cancer evolution and drug resistance, emerging evidence shows that a fraction of cancer cells are never targeted at treatment onset and are thus most likely responsible for relapses.^[^ ^3^ ^]^ Indeed, one of the most important recent advances in cancer biology is the discovery that there are multiple subpopulations of cancer cells with distinct morphology, molecular profile, and pharmacological sensitivity, not only among patients but also within the same tumor.^[^ ^4^, ^5^ ^]^ Such cellular diversity, commonly known as tumor heterogeneity, is believed to be a main driver for cancer evolution and poses a critical challenge to the efficacy of targeted therapies, and precision or individualized medicine.^[^ ^6^, ^7^ ^]^

Although theoretically additional drugs could be used together to cover all cancer cell subpopulations, this may be hard to effectively achieve considering the increasingly complex heterogeneity, depending on the subtyping methods used, and ‌side‐effects, which are often cumulative and thus could be overly toxic.^[^ ^8^ ^]^ Importantly, cellular heterogeneity exists decades before cancer may arise.^[^ ^9^, ^10^ ^]^ This suggests that the presence of multiple cell subtypes is not only a normal aspect of organ physiology, but they are likely to depend on and interact with each other throughout organ development and disease progression. Thus, one potential solution to covering all cancer cell populations with a minimum number of drugs is to target, rather than each of the individual subgroups, their mutual interactions and dependence. To do so, however, we must first understand the mechanism by which subgroups interact with each other.

As a powerful model amenable to experimental manipulations, the mouse mammary gland is well known for its epithelial and stromal heterogeneity in development and breast cancer^[^ ^11^, ^12^, ^13^, ^14^ ^]^ and, thus, could be an ideal system to interrogate the molecular basis of subgroup interactions. Indeed, regardless of their molecular heterogeneity, all breast cancer cells can be traced back to only two cell types, namely luminal and basal cells. Both are derived from embryonic mammary stem cells (MaSCs) and form the mammary gland via epithelial branching morphogenesis, which is slow from around embryonic day (E) 16.5 to 3 weeks at puberty, but is fast and rigorous during the following 6–7 weeks until a mature mammary gland forms upon reaching adulthood.^[^ ^15^ ^]^ Interestingly, although these two lineages are separated by birth, basal cells can become luminal cells when, for example, they are transplanted into the cleared fat‐pad of a female mouse or luminal cells are otherwise under‐represented in the mammary epithelium, leading to the belief that there are adult MaSCs in the basal compartment.^[^ ^16^, ^17^, ^18^ ^]^

Importantly, differentiation and branching morphogenesis of mammary epithelium depend on its interactions with the stromal microenvironment, including fibroblasts and the extracellular matrix (ECM).^[^ ^19^, ^20^, ^21^, ^22^ ^]^ We showed that fibroblast production of FGF ligands, especially FGF10, regulates epithelial migration, branching, and expansion.^[^ ^23^, ^24^ ^]^ Although basal cells and luminal cells collaborate during mammary epithelial branching, its molecular basis has remained largely unknown.^[^ ^25^, ^26^, ^27^ ^]^ Here, we hypothesize that fibroblast FGFs regulate basal‐luminal interactions essential for epithelial branching and cancer progression. We tested this hypothesis by first examining the consequence of the loss of FGF signaling in basal cells or luminal cells.

## 2 Results

### 2.1 *Fgfr2* Function in Basal Cells Is Required for Mammary Gland Regeneration

**Figure 1:**
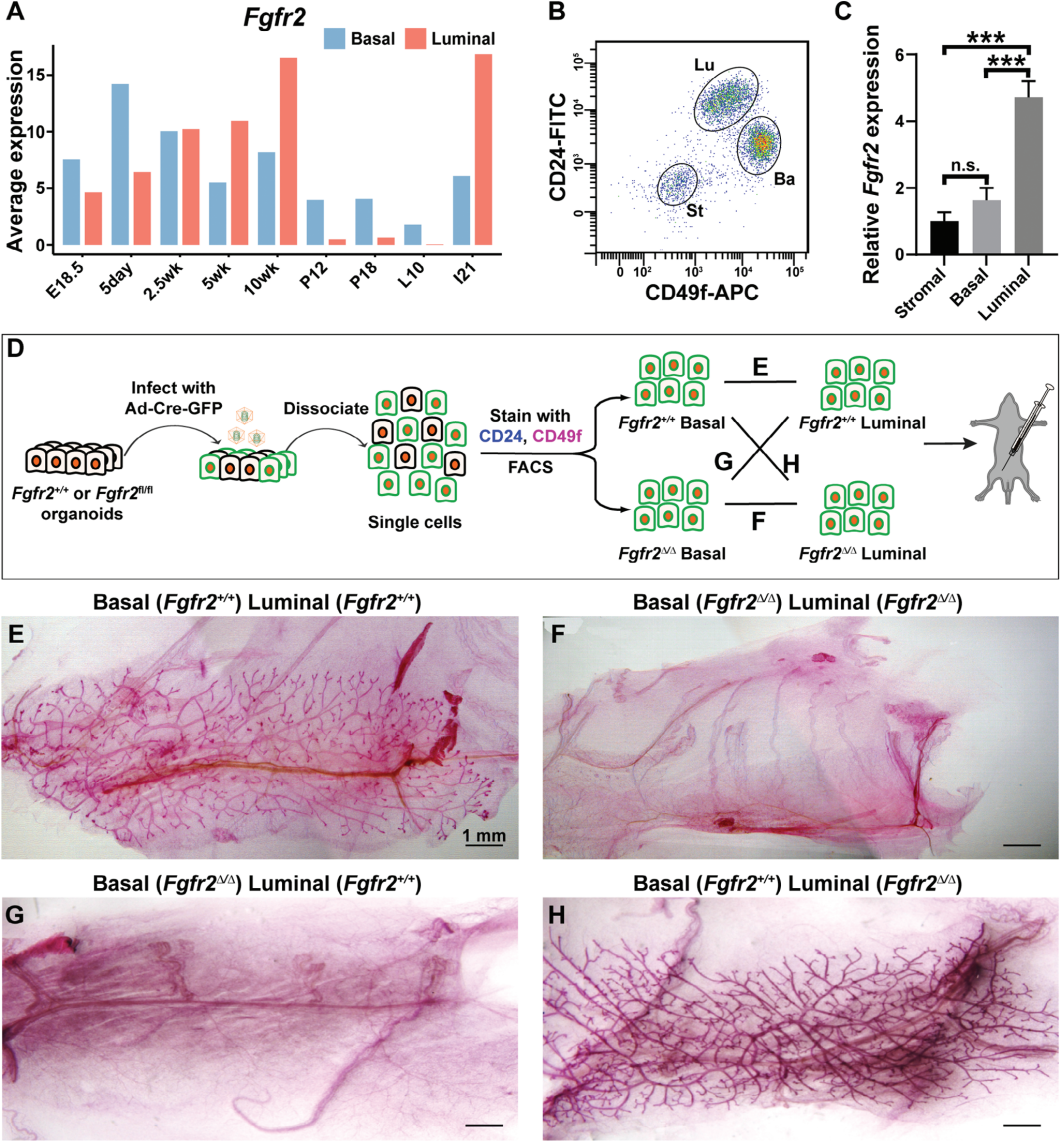
*Fgfr2* function in basal cells is required for mammary gland regeneration. A) mRNA expression of *Fgfr2* in basal and luminal cells at different stages of mouse mammary gland development based on scRNA‐seq. B) Gating strategy for sorting of mammary gland cell populations based on CD24 and Itga6 (CD49f) by FACS. CD24^neg^CD49f^neg^ were stromal cells (St), CD24^hi^CD49f^low^ were luminal cells (Lu), CD24^med^CD49f^hi^ were basal cells (Ba). C) Relative expression of *Fgfr2* in sorted luminal, basal, and stromal cells. D) Schematic diagram depicting the experimental procedure of sample preparation, adenoviral infection, FACS, and transplantation strategy. Approximately 20 000 cells were injected into the cleared fat pad. E–H) Epithelial network as revealed by Carmine‐staining in recombinant mammary glands derived from E) wild‐type basal and luminal cells, F) *Fgfr2* ^∆/∆^ null basal and luminal cells, G) *Fgfr2* ^∆/∆^ null basal and wild‐type luminal cells, H) wild‐type basal and *Fgfr2* ^∆/∆^ null luminal cells. At least three transplants from each group were analyzed. Scale bars: 1000 µm.

Using published databases of single‐cell RNA (scRNA) transcriptomics of the developing mouse mammary gland,^[^ ^28^ ^]^ we found *Fgfr2* is expressed in both basal and luminal epithelial cells with a dynamic luminal‐to‐basal ratio during mammary development, including during branching morphogenesis at the prepubertal 5‐day and 2.5‐week stages, and post‐pubertal 5‐week and 10‐week stages, pregnancy, lactation, and involution stages (**Figure** **1**A). To validate these results, we prepared cDNA templates from the basal, luminal, and stromal cells of 8‐week‐old female mice and performed qPCR reactions. We found that *Fgfr2* mRNA is present in both basal and stromal cells, but its level is highest in luminal cells (Figure 1B,C), confirming the scRNA data at this stage.

The observation that *Fgfr2* mRNA expression is in luminal and basal cells suggests it plays roles in these two cell types. To test this possibility, we sought to examine the consequences of *Fgfr2* loss in luminal and basal cells. We isolated mammary epithelial cells (MECs) from *Fgfr2* ^+/+^ and *Fgfr2* ^fl/fl^ mice and infected them with adenovirus‐Cre‐GFP to generate control (*Fgfr2* ^+/+^) and mutant (*Fgfr2* ^∆/∆^) cells, respectively, and sorted for basal and luminal cells (Figure 1D). We then mixed sorted cells in such a way that *Fgfr2* was present in (Figure 1E) or absent from (Figure 1F) both luminal and basal cells; or it was absent from only basal cells (Figure 1G) or only luminal cells (Figure 1H) before we transplanted MEC mixtures into the cleared fat‐pads of immune‐deficient nude mice. Transplants were harvested 8 weeks post‐surgery and were examined by whole‐mount Carmine‐staining to reveal the epithelial network. We confirmed that the simultaneous loss of *Fgfr2* in both the basal and luminal compartments resulted in a complete failure of mammary gland regeneration (Figure 1E,F). However, mammary regeneration failed when *Fgfr2* was specifically removed from basal cells (Figure 1G) but was successful when *Fgfr2* was removed only from luminal cells (Figure 1H).

Together, the data suggest that *Fgfr2* is required in the basal, but not luminal cells, for mammary gland regeneration, presumably because of its different functions in these two cell types.

### 2.2 *Fgfr2* Promotes Luminal Epithelial Branching and Expansion during Mammary Gland Regeneration

One possibility for the above intriguing phenotype is that different isoforms, namely the IIIb and IIIc isoforms known to have differential affinities to FGF ligands and downstream effectors,^[^ ^29^ ^]^ of FGFR2 may exist in these two epithelial cell types. Using scRNA transcriptomics, we confirmed our previous study showing that multiple *Fgf* ligands, including *Fgf2*, *Fgf7*, and *Fgf10* among others^[^ ^25^ ^]^ are expressed by mammary gland fibroblasts (Figure S1A–C, Supporting Information). However, both the *Fgfr2 IIIb* and *IIIc* isoforms are similarly expressed by basal and luminal cells (Figure S1D,E, Supporting Information), thus ruling out the possibility that their differential expression causes the distinct phenotypes observed due to *Fgfr2* loss in basal and luminal cells.

Another possibility is that the luminal cells in the regenerated mammary gland were actually wild type, as they may have been derived from wild‐type basal stem cells when *Fgfr2* null luminal cells died or otherwise failed to expand. To examine this possibility, we tracked the fate of *Fgfr2* ^∆/∆^ luminal cells using the *R26R* ^mG^ allele.^[^ ^30^ ^]^ Thus, we isolated *Fgfr2* ^+/+^; *R26R* ^mG^ and *Fgfr2* ^fl/fl^; *R26R* ^mG^ MECs and infected them with adenovirus‐Cre‐GFP. *Fgfr2* ^+/+^; *R26R* ^mG^ basal cells, which were red, were then sorted and mixed with either control *Fgfr2* ^+/+^; *R26R* ^mG^ or mutant *Fgfr2* ^∆/∆^; *R26R* ^mG^ luminal cells, both of which were green, before we transplanted the mixture into the cleared fat‐pads of nude mice (Figure S2A, Supporting Information).

Eight weeks after transplantation, we examined the epithelial networks from the control and experimental glands under a fluorescent stereoscope (Figure S2B–E, Supporting Information). We found that wild‐type luminal cells contributed to the entire epithelial network in the control gland (Figure S2B,C, Supporting Information), but *Fgfr2* null luminal cells made up only a tiny portion of the experimental gland (Figure S2D,E, Supporting Information). Although K8^+^ luminal cells were green in the control gland, consistent with the notion that they were derived from the green luminal donor cells (Figure S2F–F’’’, Supporting Information), they were instead red in the experimental gland, suggesting that they were derivatives of the red basal donor cells (Figure S2G–G’’’, Supporting Information). Indeed, the tdTomato fluorescence was much brighter in the experimental gland than in the control gland (Figure S2B,D, Supporting Information), again consistent with the notion that there were more red fluorescent cells in the former than in the latter gland.

The presence of *Fgfr2* null luminal cells 8‐weeks after transplantation and their contribution, albeit limited, to the epithelial tree, argue against the possibility that *Fgfr2* functions as a survival factor; rather, they suggest *Fgfr2* plays a role in luminal expansion and branching. Therefore, we isolated *Fgfr2* ^+/+^ and *Fgfr2* ^∆/∆^ luminal cells and directly tested their abilities to form colonies and undergo branching. We found that *Fgfr2* ^∆/∆^ luminal cells had a three‐fold reduction in colony‐forming ability when compared with wild‐type cells (Figure S3A, Supporting Information). Moreover, whereas wild‐type luminal cells could form many (≈4–6) branches in medium containing FGF2, *Fgfr2* ^∆/∆^ luminal cells could barely do so even in the highest FGF2 concentration tested (Figure S3B–D, Supporting Information). Together, the data show that *Fgfr2* is required for epithelial branching morphogenesis and expansion of the luminal epithelium.

### 2.3 Stromal FGFs Are Essential for Mammary Stem Cell Pool Expansion, But Not Differentiation or Survival

**Figure 2:**
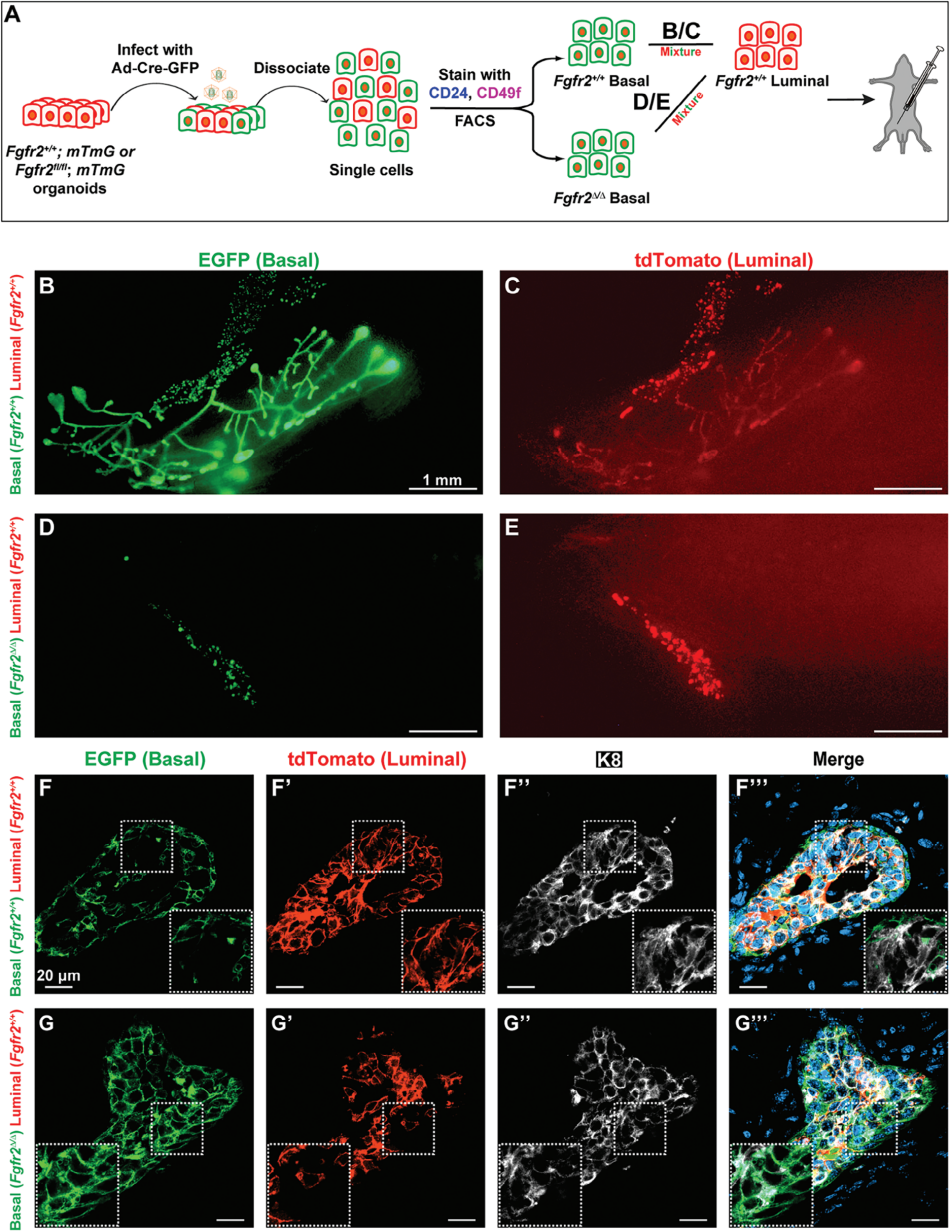
Stromal FGFs are essential for mammary stem cell pool expansion, but not differentiation or survival. A) Schematic diagram depicting the experimental procedure of sample preparation, adenoviral infection, FACS, and transplantation strategy. Approximately 20 000 cells were injected into the cleared fat pad. B–E) Whole‐mount or immunofluorescence on frozen sections F–G’’’) of the epithelial network 2 weeks after transplantation. The EGFP channel allowed visualization of epithelial cells derived from green wild‐type B) and *Fgfr2* ^∆/∆^ null D) basal cells and the tdTomato channel allowed visualization of red wild‐type luminal cells (C,E). Note that a few wild‐type basal cells gave rise to luminal cells in the middle of the epithelium (inset in F–F’’’), while many *Fgfr2* null basal cells also differentiated into luminal cells as they were K8+ (G’’).

We also wondered about the fate of *Fgfr2* ^∆/∆^ basal cells, which failed to regenerate the mammary gland when transplanted together with wild‐type luminal cells (Figure 1G). Using a similar strategy as above, we marked *Fgfr2* ^+/+^; *R26R* ^mG^ and *Fgfr2* ^∆/∆^; *R26R* ^mG^ basal cells green and separately mixed them with red *Fgfr2* ^+/+^; *R26R* ^mG^ luminal cells before we transplanted the mixture (**Figure** **2**A). As expected, a complete mammary tree was regenerated only when red wild‐type luminal cells were mixed with green wild‐type basal cells, both of which contributed to the entire network (Figure 2B,C), but not with green *Fgfr2* ^∆/∆^ basal cells, both of which were restricted to the injection site (Figure 2D,E). When frozen sectioned and examined under a confocal microscope, green *Fgfr2* ^+/+^ basal cells of the control gland stayed mostly as basal cells on the outside of the epithelium (Figure 2F,F’), except for a few cells in the middle, where they had become luminal (Figure 2F’–F’’’). By contrast, although a complete gland failed to form, many green *Fgfr2* ^∆/∆^ cells of the experimental gland were found in the middle of the small epithelial growths where they were surrounded by derivatives of the red donor luminal cells (Figure 2G–G’’’), showing that *Fgfr2* null basal cells had adopted a luminal fate, thus suggesting that *Fgfr2* function is not required for luminal differentiation.

The data thus suggest that *Fgfr2* is required for expansion, rather than for survivor or differentiation of basal stem cells. In the absence of *Fgfr2*, basal cells cannot expand, and mammary gland regeneration does not occur. Together, the data show that both basal and luminal cells strictly depend on stromal FGFs to expand, thus highlighting stromal‐to‐basal/luminal FGF signaling as a potential target to limit epithelial growths. Finally, the presence of both luminal and basal cells when *Fgfr2* is removed could be explained by the presence of *Fgfr1* in the mammary epithelium,^[^ ^31^ ^]^ which may play a redundant role as *Fgfr2*.

### 2.4 Expansion of Mammary Stem Cells Depends on Luminal Paracrine Factors

**Figure 3:**
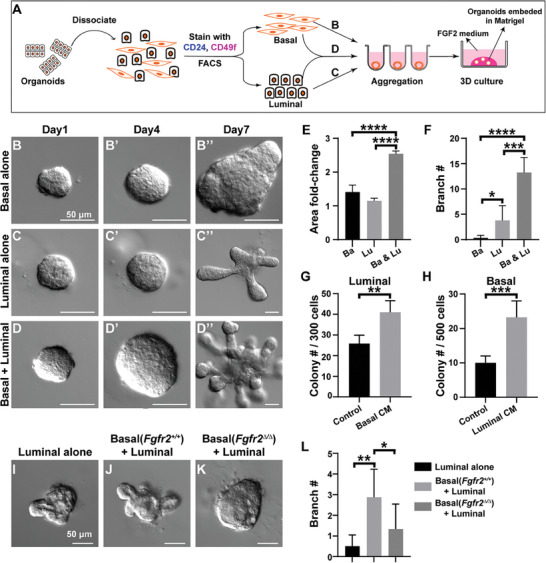
Expansion of mammary stem cells depends on luminal paracrine factors. A) Schematic diagram depicting the experimental procedures of organoid preparation, basal and luminal cell sorting, aggregation and in vitro culture methods. Approximately 1000 cells were used to form each aggregate. B–F) in vitro assays in which purified cells were either cultured separately as B–B’’) basal cells, C–C’’) luminal cells, or D–D’’) a mixture containing both basal and luminal at a 1:1 ratio. Aggregate growths, as measured by fold changes of their areas over E) a 4‐day culture and by the number of branches they formed after F) a 7‐day culture, were quantified. G,H) Quantitative analysis of colony‐forming efficiency by wild‐type luminal or basal cells cultured in control medium or conditioned medium from G) the basal or H) luminal cells, respectively. Note that the conditioned medium (CM) from both basal and luminal cells could stimulate growths of the other cell type, suggesting that growth stimulation was, at least partially paracrine in nature. I–L) Basal‐luminal co‐culture experiments in which wild‐type luminal cells were cultured alone (I), or together with either J) wild‐type or K) *Fgfr2* ^∆/∆^ null basal cells. Seven days after culture their branch numbers were quantified (L). Values shown are the mean ± SD for each data point: *P < 0.05; **P < 0.01; ***P < 0.001; ****P < 0.0001. unpaired, two‐tailed Student's *t* tests. Scale bars: 50 µm. At least 6 aggregates were used for each data point.

It is interesting that wild‐type luminal epithelium cannot expand in the presence of basal epithelium lacking *Fgfr2*. One possibility is that luminal expansion requires basal factors that are regulated by *Fgfr2* function in basal cells. To facilitate the determination of the molecular basis on which basal stem cells and luminal cells interact, we first sought to set up an in vitro system so their interactions can be accurately measured. We harvested basal and luminal cells from 8‐week‐old mammary glands, aggregated them either separately or together, and embedded them in Matrigel. The aggregates were then cultured in FGF2 basic medium for four days when their growths were measured or for seven days when their number of branches was counted (**Figure** **3**A).

We found that aggregates containing either basal or luminal cells alone grew somewhat in size, but those containing both basal and luminal cells grew significantly more at two to three‐fold over a four‐day culture (Figure 3B–E). Likewise, while luminal aggregates, but not basal aggregates, could form a few branches (≈4) after culture, those containing both basal and luminal cells formed branches most readily (≈13) (Figure 3B–D’’,F). These data suggest that the in vitro culture system could mimic in vivo basal‐luminal reciprocal stimulations. Moreover, we found that the conditioned medium from basal cells or luminal cells could increase colony‐forming ability of luminal or basal aggregates by ≈48% or ≈120%, respectively (Figure 3G,H). The data thus suggest that basal‐luminal interactions are, at least partially, paracrine in nature.

Using this system, we next determined the consequence of *Fgfr2* loss in basal stem cells on basal‐to‐luminal stimulation. We found wild‐type basal cells promoted luminal cell expansion by approximately six‐fold (Figure 3I–L); however, removal of *Fgfr2* from basal cells reduced the expansion by nearly 50% (Figure 3J–L), a result that is consistent with what we observed in vivo. Together, we concluded that the in vitro system relatively accurately mimics the in vivo process and could help identify candidate factors that mediate basal–luminal interactions.

### 2.5 BMP7 Is a Basal‐to‐Luminal Paracrine Candidate Regulated by Stromal FGFs

**Figure 4:**
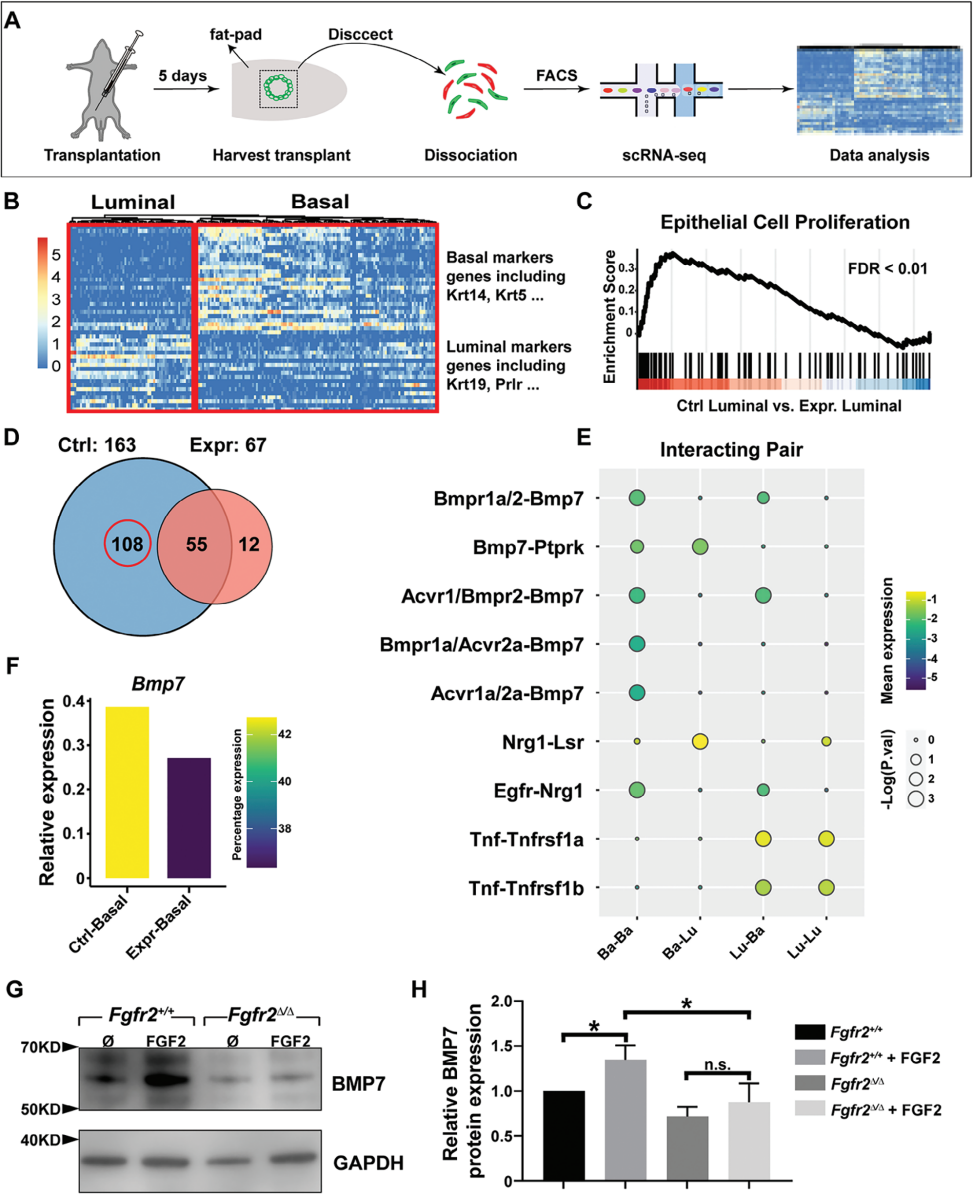
BMP7 is a basal‐to‐luminal paracrine candidate regulated by stromal FGFs. A) Diagram depicting the experimental procedures of single‐cell analyses of transplants from mixtures of wild‐type luminal cells with either wild‐type or *Fgfr2* ^∆/∆^ null basal cells five days after surgery. B) Identification of basal and luminal cells were based on expression of published marker gene sets for these two cell types.^[^ ^48^ ^]^ C) GSEA analysis of pathways related to epithelial cell proliferation in the luminal cells of the control group and the experimental group, in which basal cells were wild type or *Fgfr2* ^∆/∆^ null cells, respectively. D) Comparison of the number of ligand‐receptor pairs between basal and luminal cells based on CellphoneDB analysis in control and experimental samples. E) Select ligand‐receptor pairs between basal and luminal cells based on CellphoneDB analysis. F) *Bmp7* mRNA expression in control (*Fgfr2* ^+/+^) and experimental (*Fgfr2* ^∆/∆^) basal cells, confirming its reduced experiment in mutant basal cells. G) Western blotting and H) quantification analysis of BMP7 and the internal control GAPDH protein in the absence or presence of FGF2 stimulation using wild‐type and *Fgfr2* null primary mammary epithelial basal cells. Ø denotes control group without FGF2 addition. Graph shows mean ± SD. Unpaired Student's t‐test was performed for statistical analysis, n.s., not significant, *P* ≥ 0.05; *P < 0.05.

We sought to use transcriptomics to determine the paracrine factors by which basal cells promote luminal expansion. We transplanted a mixture of red *Fgfr2* ^+/+^; *R26R* ^mG^ luminal cells with either green *Fgfr2* ^+/+^; *R26R* ^mG^ or *Fgfr2* ^∆/∆^; *R26R* ^mG^ basal cells into cleared fat‐pads of 3‐week‐old female nude mice. Transplanted cells were dissected out under a fluorescent stereoscope five days post‐surgery, purified via FACS, and subjected to scRNA‐seq procedures using the 10X Genomic platform (**Figure** **4**A). After quality control and removal of immune cells from a total population of cells, whose transcriptomes were successfully sequenced^[^ ^32^ ^]^ (see Experimental Section for details), we separated them into basal and luminal populations using published panels of marker genes (Figure 4B).^[^ ^33^ ^]^

Using bioinformatics analyses, we determined differentially expressed genes (DEGs) between control and experimental basal cells (Figure S4A, Supporting Information) and luminal cells (Figure S4B, Supporting Information). We discovered pathways whose component genes were up‐regulated in the experimental basal cells (Figure S4C, Supporting Information) and luminal cells (Figure S4D, Supporting Information) when compared with those in the control groups. Consistent with our prediction that *Fgfr2* loss reduces basal‐to‐luminal promotion, GSEA analysis showed that the expression of cell proliferation marker genes was higher in control luminal cells than experimental luminal cells, both of which were wild type (Figure 4C).

We used CellphoneDB, which defines highly significant ligand‐receptor interactions between different cell populations in single‐cell data^[^ ^34^ ^]^ and found 163 and 67 significant ligand‐receptor‐pair interactions in control and experimental groups, respectively, with 108 pairs uniquely present in the control group (Figure 4D) (Table S2, Supporting Information). To shorten the list of candidate genes, we considered ligands and receptors that were highly expressed in basal cells and luminal cells, respectively. Given that *Bmp7* was the most heavily represented among the uniquely expressed basal ligands (Figure 4E) and that its expression was indeed reduced in *Fgfr2* null basal cells when compared with wild‐type basal cells from the scRNA‐seq data (Figure 4F), we focused on *Bmp7* as the candidate basal factor in this study.

Thus, we first determined whether FGF signaling regulates BMP7 expression. Using qPCR, we found FGF2 increased mRNA expression of *Etv5*, a known FGF signaling target, and *Bmp7*, by ≈eight‐fold and two‐fold, respectively (Figure S5A, Supporting Information). Western blotting analysis confirmed that FGF2 stimulation increased BMP7 protein expression by 50% (Figure S5B, C, Supporting Information). In contrast, loss of FGF signaling activities appeared to have an opposite effect on *Bmp7* expression. Specifically, the addition of SU5402 or BGJ398, an FGFR inhibitor, greatly reduced mRNA expression of *Etv5* and *Bmp7* in a medium containing FGF2 (Figure S5D,E, Supporting Information). Likewise, the upregulation of both *Etv5* and *Bmp7* mRNA expression because of FGF2 stimulation was completely offset by the removal of *Fgfr2* function at both the protein level as detected by Western blotting (Figure 4G,H) or the transcriptional level as detected by qPCR (Figure S5F, Supporting Information). Finally, FGF10 and FGF7 could increase the mRNA expression of the FGF signaling target *Etv4* and *Bmp7* (Figure S5G, Supporting Information), suggesting that overall stromal FGFs have similar promoting effects on epithelial BMP signaling.

Together, these data confirmed that, besides promoting basal expansion, stromal‐to‐epithelial FGFR2 signaling also regulates basal production of BMP7, a candidate paracrine factor required for luminal epithelial expansion.

### 2.6 Basal Derived BMP7 Promotes Luminal Expansion

**Figure 5:**
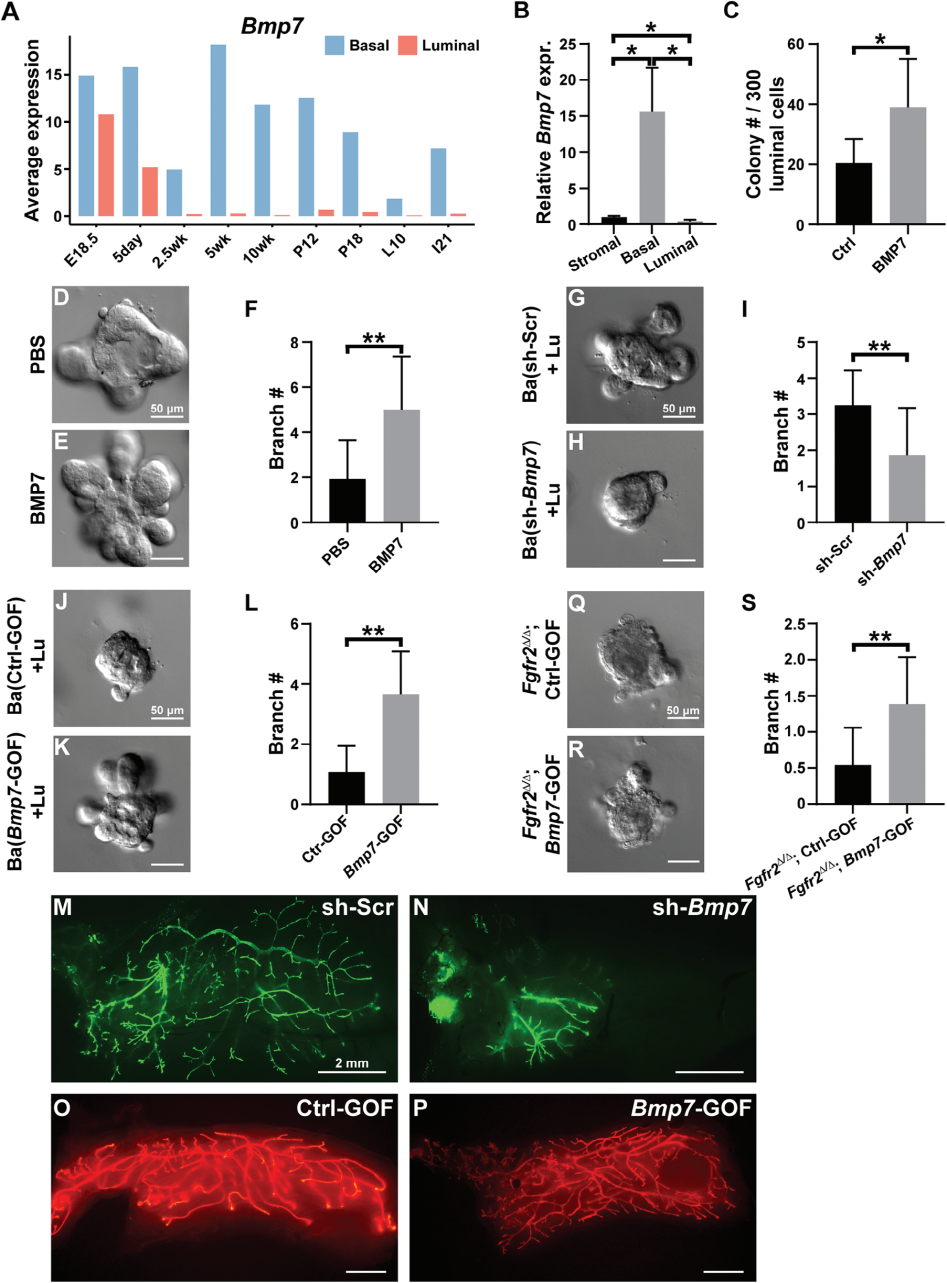
Basal derived BMP7 promotes luminal expansion. A) mRNA expression of *Bmp7* in basal and luminal cells at different stages of mouse mammary gland development based on mining scRNA‐seq datasets. B) Relative expression of *Bmp7* in sorted luminal, basal, and stromal cells. C) Quantification of colonies formed by luminal cells cultured in medium containing with either control PBS or 50 ng mL^−1^ BMP7. D–F) Luminal cells were cultured with D) either PBS or E) BMP7 added to the medium, with branch number quantified 7 d later (F). G–I) Wild‐type luminal cells were cultured with basal cells transfected with a lentiviral construct expressing G) either control shRNA or H) sh‐*Bmp7*, with branch number quantified several days later (I). J–L) Wild‐type luminal cells were cultured with basal cells transfected with a lentiviral construct of either J) a control or K) excessive *Bmp7* expression, with branch number quantified seven days later (L). M–P) Wholemount mammary gland epithelial network observed under a fluorescent stereoscope 8 weeks after transplantation of M,N) MECs carrying lentiviral shRNAs or O,P) overexpression constructs. At least three glands were used for each data point. Q–S) Branching assay of wild‐type luminal cells cultured together with *Fgfr2* ^∆/∆^ null basal cells over‐expressing the mRNA of either a Q) control or R) *Bmp7*. Consistent with *Bmp7* working downstream of *Fgfr2*, the ability of luminal cells to form branches was increased by approximately three‐fold due to S) *Bmp7* overexpression. At least 9 aggregates were used for each data point. Values shown are the mean ± SD for each data point. n.s., not significant, *P < 0.05; ** *P* < 0.01. unpaired, two‐tailed Student's *t* tests. Scale bars: 50 µm.

Using the published scRNA‐seq databases mentioned above, we found that *Bmp7* is enriched in basal cells during mammary gland development, especially around puberty onset at two‐and‐a‐half weeks of age when it is almost exclusively expressed by basal cells (**Figure** **5**A). We performed qPCR and confirmed that *Bmp7* is specifically enriched in basal stem cells of the 8‐week‐old mammary gland (Figure 5B), during active epithelial branching and expansion. Moreover, the addition of BMP7 to the medium increased the ability of luminal cells to form colonies by ≈two‐fold (Figures 5C and S6A,B, Supporting Information), or to undergo branching by ≈2.5‐fold (Figure 5D–F).

Next, we used both loss‐ and gain‐of‐function approaches to directly test how basal *Bmp7* affected luminal branching. We found a lentiviral construct expressing *Bmp7* shRNA (sh‐*Bmp7*) reduced its mRNA expression by ≈50% (Figure S6C, Supporting Information). As expected, *Bmp7* reduction did not affect colony formation by basal cells (Figure S6D, Supporting Information). However, consistent with being targeted by *Bmp7*, luminal cells showed a reduced ability to undergo branching by ≈40% when they were co‐cultured with basal cells expressing *sh‐Bmp7* instead of the scramble control *sh‐Scr* (Figure 5G–I).

Using an overexpression vector, we increased *Bmp7* mRNA expression by over eight‐fold, resulting in a 15% increase in the colony‐forming ability of basal cells (Figure S6E,F, Supporting Information). The effects on luminal cells were more pronounced, showing a ≈three‐fold increase in their ability to undergo branching when cocultured with *Bmp7*‐GOF basal cells compared to control basal cells (Figure 5J–L).

We then sought to validate the above in vitro data using the transplantation model. Thus, primary MECs were transfected with either control sh‐*Scr* or the experimental sh‐*Bmp7* construct from the above LOF experiment before we transplanted them. We found that sh‐*Bmp7*‐mediated knockdown greatly reduced epithelial regeneration (Figure 5M,N). Consistent with this result, cell proliferation, as marked by Ki67 staining, was reduced by ≈70% in luminal cells of the experimental transplants where *Bmp7* expression was knocked down in epithelial cells (Figure S6G–I, Supporting Information). By contrast, forceful expression of *Bmp7* in basal cells greatly increased branching in the experimental gland when compared with the control gland (Figure 5O,P).

Finally, we asked whether forceful expression of *Bmp7* could compensate for *Fgfr2* loss in basal cells. If so, we predicted that *Bmp7* GOF would rescue the *Fgfr2* LOF defects. To answer this question, we transfected *Fgfr2* null basal cells with the *Bmp7*‐GOF construct and cultured them together with wild‐type luminal cells. We found that *Bmp7* GOF in basal cells rescued the branching defect of the experimental luminal cells by nearly two‐fold (Figure 5Q–S). However, *Bmp7* GOF failed to rescue the mammary gland regeneration defect due to *Fgfr2* loss in basal cells in the in vivo transplantation assay (Figure S6J–L, Supporting Information), suggesting that *Bmp7* is not the only *Fgfr2* target required for the regeneration process.

Together, our data show that *Bmp7* functions downstream of *Fgfr2* in basal cells, promoting luminal cell proliferation and expansion in a paracrine manner during mammary epithelial branching.

### 2.7 BMP7‐BMPR1a/2 Signaling Mediates Basal‐to‐Luminal Promotion of Epithelial Proliferation and Expansion In Vivo

Consistent with the prediction that basal *Bmp7* targets luminal cells, luminal cells expressed both of its cognate heterodimer receptors, *Bmpr1a* and *Bmpr2*, although they were also expressed by basal cells (Figure S7A,B, Supporting Information). Using qPCR, we confirmed that these two receptors were expressed by basal and luminal cells of the 8‐week‐old mammary gland (Figure S7C,D, Supporting Information). To directly examine the function of the BMP receptors in luminal expansion, we constructed shRNA‐expression vectors and found that they reduced *Bmpr1a* and *Bmpr2* mRNA expression levels by ≈80% and 70%, respectively, in the luminal line of HC11 cells, as measured by qPCR (Figure S7E,F, Supporting Information). Due to *Bmpr1a* and *Bmpr2* knockdown, HC11 cell numbers were reduced by ≈20% and 30% on day 1, respectively, and we also observed a reduction on day 2 (Figure S7G, Supporting Information). Overexpression of *Bmpr1a* and *Bmpr2* led to a thirteen‐fold and a three‐fold increase in mRNA expression of each gene (Figure S7H,I, Supporting Information). However, only *Bmpr2‐*GOF increased HC11 cell number by day 1 and no significant changes were observed under any other conditions (Figure S7J, Supporting Information).

In addition, consistent with the prediction that *Bmpr1a* and *Bmpr2* promote luminal expansion, the expression of sh*‐Bmpr1a* or sh*‐Bmpr2* reduced the colony‐forming ability of primary luminal cells by ≈40% and 30%, respectively (Figure S7K–N, Supporting Information). As predicted for a ligand of BMP signaling, BMP7 stimulation triggered the expression of phosphor‐SMAD1/5, an activated form of a signaling downstream component (Figure S8A, Supporting Information). Likewise, mRNA expression of *Id1* and *Id2*, both of which are BMP signaling target genes, was increased by over ten‐fold in luminal cells by BMP7 stimulation (Figure S8B, Supporting Information). The expression of sh‐*Bmpr1a* or/and sh‐*Bmpr2* only modestly reduced phosphor‐SMAD1/5 and mRNA expression of the target genes *Id1*, *Id2*, and *Jun* in HC11 cells, as shown by Western Blotting and qPCR results, respectively (Figure S8C–E, Supporting Information).

Together, these data show that BMP7‐BMPR signaling promotes luminal epithelial expansion.

### 2.8 Luminal INHBA Is a BMP7 Signaling Target That Promotes Expansion of Basal Stem Cell Pool

**Figure 6:**
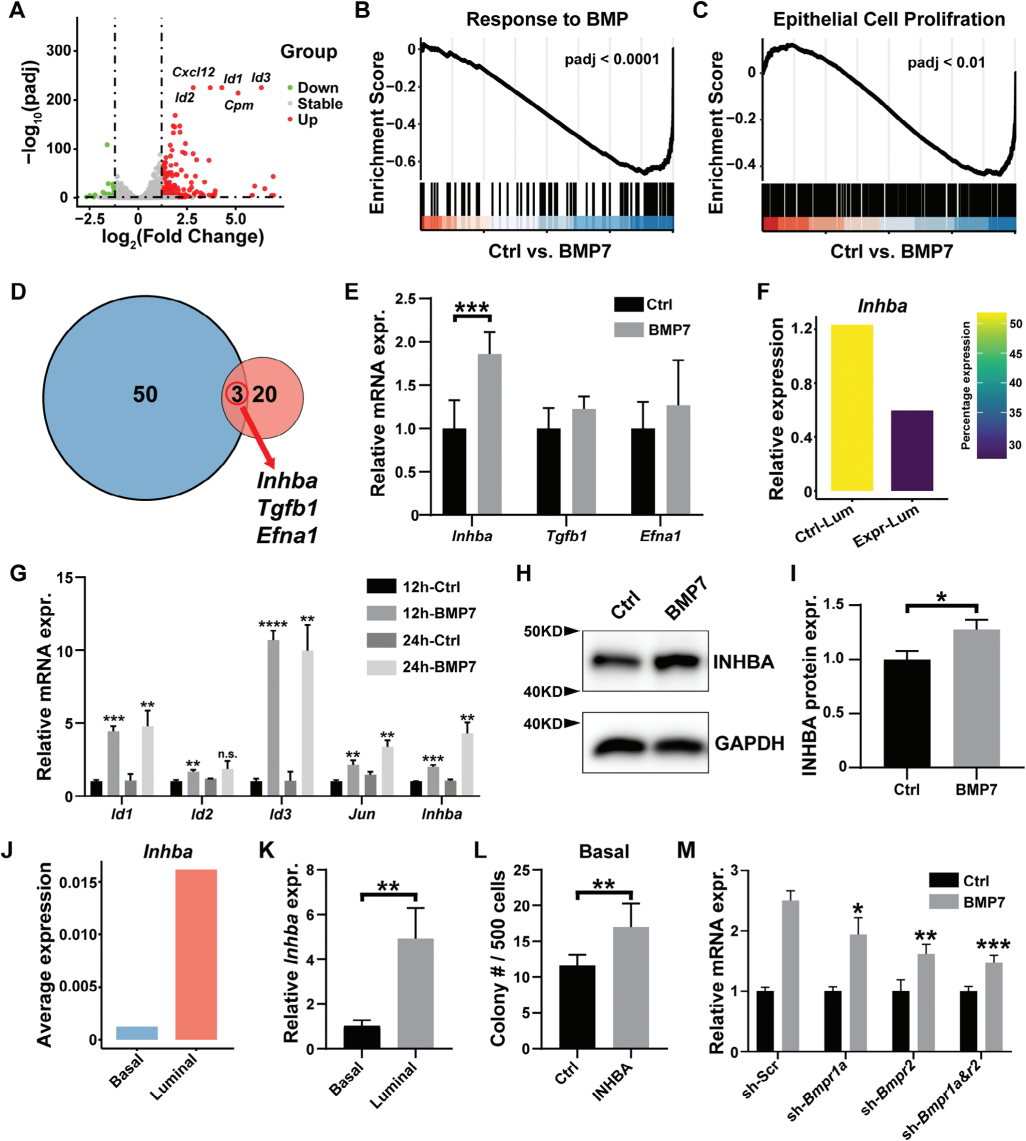
Luminal INHBA is a BMP7 signaling target that promotes expansion of basal stem cell pool. A) Differentially expressed genes in response to BMP7 stimulation, with those significantly (fold change >1.2, adjusted‐*p*‐value <0.05) upregulated genes marked red and down‐regulated genes marked green. Note that several known BMP signaling target genes, including *Id1*, *2*, and *3* were among the upregulated ones. B,C) GSEA analysis of B) the BMP signaling pathway and C) cell proliferation pathway. Both of these two pathways were upregulated in HC11 cells stimulated by BMP7 when compared with control. D) Venn diagram analysis of the 50 upregulated genes in response to BMP7 stimulation and the 20 luminal‐to‐basal ligand‐receptor pairs described above (Figure 4D). E) mRNA expression as detected by qPCR of the BMP7 candidate targets by luminal cells cultured in medium with or without BMP7 stimulation. F) *Inhba* mRNA expression in luminal cells of transplants derived from wild‐type luminal cells coinjected with either control (*Fgfr2* ^+/+^) and experimental (*Fgfr2* ^∆/∆^) basal cells as described above (Figure 4A). Note that its expression is greatly reduced in the experimental transplants. G) Relative mRNA expression of the BMP signaling target genes and *Inhba* in luminal cells cultured in medium with or without 50 ng mL^−1^ BMP7 for the durations indicated. H) Western blotting and I) quantification analysis of INHBA and the internal control GAPDH in the absence or presence of BMP7 stimulation. Note that BMP7 significantly upregulated INHBA protein expression. J) mRNA expression of *Inhba* in the 8‐week developing mammary gland using the above scRNA‐seq datasets from this stage. K) Validation of relative *Inhba* mRNA expression in sorted luminal and basal cells at the 8‐week stage using qPCR. L) Quantification of colonies formed by 500 basal cells cultured in medium with or without 5 ng mL^−1^ INHBA. M) qPCR examination of the relative mRNA expression of *Inhba* in the presence of BMP7 stimulation in luminal cells expressing *Bmpr1a* and *Bmpr2* shRNAs, either individually or in combination. Values shown are the mean ± SD for each data point: *P < 0.05; **P < 0.01; ***P < 0.001; ****P < 0.0001. Unpaired, two‐tailed Student's *t* tests.

Having determined the basis of basal‐to‐luminal interactions, we next sought to identify the nature of the paracrine factor mediating luminal‐to‐basal promotion under the control of BMP7 signaling. Therefore, we stimulated the HC11 cells using a medium with or without BMP7 protein before subjecting them to procedures of RNA‐sequencing. As expected, we found that BMP7 stimulation increased the expression of a panel of genes, including *Id1*, *Id2*, and *Id3* (**Figure** **6**A). Likewise, GSEA analysis showed that genes associated with BMP signaling (Figure 6B) and epithelial cell proliferation (Figure 6C) were upregulated in BMP7‐stimulated cell group, as expected. To narrow down the list of candidates of BMP7 target genes, we picked the top 50 ligand genes that were specifically upregulated by BMP7 stimulation and focused on those three, namely *Inhba*, *Tgfb1*, and *Efna1*, which were also among the top 20 luminal‐basal ligand‐receptor pairs discovered by the CellphoneDB analysis mentioned above (Figure 6D). qPCR analysis showed that mRNA expression of only *Inhba* was upregulated by BMP7 in luminal cells, whereas the other two were not (Figure 6E). Thus, we focused on *Inhba* as a candidate paracrine factor that mediates luminal‐to‐basal interactions.

Using the above scRNA‐seq data (Figure 4A), we confirmed that *Inhba* was indeed downregulated in wild‐type luminal cells cotransplanted with *Fgfr2* null basal cells when compared with those co‐transplanted with *Fgfr2* wild‐type basal cells (Figure 6F). We also found that BMP7 stimulation increased mRNA expression of *Inhba*, as well as BMP signaling targets *Id1*, *Id2*, *Id3*, and *Jun* in primary luminal cells (Figure 6G). Moreover, using Western Blotting analysis, we found that INHBA protein expression was increased by ≈30% in the presence of BMP7 (Figure 6H,I). Together, the data confirmed our prediction that INHBA is a BMP7 target.

The above scRNA‐seq data further showed that *Inhba* is specifically expressed in luminal cells of the mammary epithelium at the 8‐week stage (Figure 6J). Using qPCR, we confirmed that the expression of *Inhba* mRNA was greatly enriched in luminal cells when compared with basal cells at this stage (Figure 6K). Using the published scRNA‐seq databases mentioned above, we also found that *Inhba* is enriched in luminal cells in the postnatal mammary gland, especially between 2.5 weeks to 10 weeks of age (Figure S9A, Supporting Information).

Moreover, adding INHBA protein to the culture medium did not affect the colony‐forming ability of luminal cells (Figure S9B, Supporting Information), but increased the colony‐forming ability of basal cells (Figure 6L) suggesting that INHBA promotes the expansion of basal cells but not luminal cells. Finally, *Inhba* upregulation at the transcriptional level, as measured by qPCR, as a result of BMP7 stimulation was compromised when *Bmpr1a* and *Bmpr2* function is inhibited by shRNA techniques against them, either individually or especially in combination (Figure 6M). The data are thus consistent with the notion that BMP signaling regulates *Inhba* expression.

Taken together, we characterized a positive feedback loop consisting of BMP7‐INHBA signaling that mediates basal and luminal interactions (Figure 8M). When a part of the loop is compromised, for example, because of the functional reduction of one or more of the feedback components, the basal stem cell pool and the luminal niche are inhibited, and mammary gland regeneration is blocked.

### 2.9 BMP7 Inhibition Blocks Triple‐Negative Breast Cancer Progression

**Figure 7:**
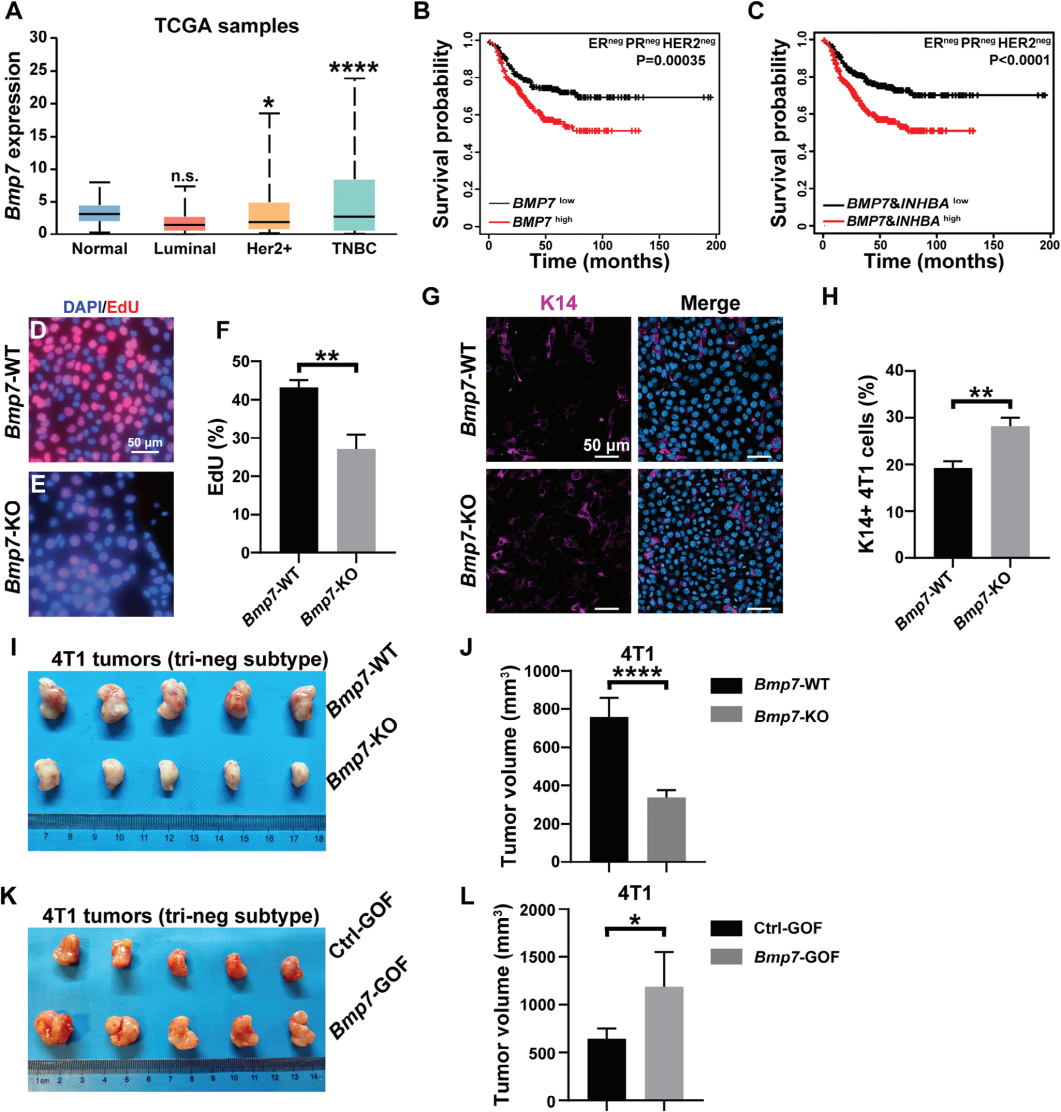
BMP7 inhibition blocks triple‐negative breast cancer progression. A) *Bmp7* mRNA expression in human breast cancer subtypes using the TCGA database. B,C) Kaplan‐Meier survival curve showing that increased expression of B) *Bmp7*, and especially C) both *Bmp7* and *Inhba* is correlated with a worse survival probability in patients within ≈150 months post‐diagnosis. D–F) Cell proliferation analysis as assessed by EdU‐incorporation in D) wild‐type and E) *Bmp7‐*KO 4T1 cells. Data are the mean ± SD. G,H) K14 immunofluorescence (red) in G) wild‐type and H) *Bmp7*‐KO 4T1 cells. Samples were costained with the nuclear dye DAPI (blue) to reveal both K14‐positive and ‐negative cells. (H) Quantification of the percentage of K14^+^ cells in the total 4T1 cell populations. I,J) Tumor growths from *Bmp7* knockout and control 4T1 cells (I), with tumor volume quantified in (J). K,L) Tumor growths from *Bmp7* gain‐of‐function and control 4T1 cells (K), with tumor volume quantified in (L). Values shown are the mean ± SD for each data point: *P < 0.05; ****P < 0.0001. unpaired, two‐tailed Student's *t* tests.

Based on the above results, we predicted that the BMP7‐INHBA signaling loop operates not only in mammary development but also in breast cancer, and that its blockage should inhibit cancer progression in different subtypes. To test this prediction, we first examined *BMP7* and *INHBA* mRNA expression in The Cancer Genome Atlas (TCGA) database. We found *BMP7* is upregulated in triple negative (ER^−^PR^−^HER2^−^) and Her2^+^ breast cancer, but not in luminal cancer (**Figure** **7**A). Moreover, increased *BMP7* expression level is positively correlated with poor survival of triple‐negative breast cancer (Figure 7B). Likewise, *INHBA* is upregulated in all three cancer subtypes and its upregulation is associated with poor survival in triple‐negative breast cancer (Figure S10A,B, Supporting Information). Interestingly, increased expression of both BMP7 and INHBA exacerbates the survival further than either gene alone, suggesting that these two genes are correlated as supported by the loop model above. Together, the data thus confirm that both *BMP7* and *INHBA* are expressed in different cancer subtypes and are likely to play a role in their progression.

Next, we used *Bmp7* as an example and tested whether its loss‐ or gain‐of‐function indeed affects the progression of triple‐negative breast cancer, where it is upregulated. To this end, we used the CRISPR technique and created a knockout clone of the 4T1 cells (Figure S10C,D, Supporting Information), a triple negative cell line.^[^ ^35^ ^]^ We then examined how *Bmp7* regulates *Inhba* mRNA expression in the 4T1 cancer cell line. We found that *Inhba* mRNA is only slightly reduced, and the change is not significant, in *Bmp7* knockout cells (Figure S10E, Supporting Information). However, *Inhba* mRNA expression is increased by ≈30% in *Bmp7‐*GOF cells. The results, thus, are not in an entire agreement with the loop that we discovered in normal development, suggesting its possible modifications in cancer settings.

Moreover, we found that *Bmp7* loss caused a ≈50% reduction in cell proliferation, as shown by EdU incorporation of 4T1 cells (Figure 7D–F). Interestingly, *Bmp7* loss caused a percentage increase of basal cells from ≈19% to 28% using the 4T1 model (Figure 7G,H), thus supporting that the strategy targets tumor heterogeneity by directly affecting luminal cells based on the above model. Furthermore, the pharmacological BMP inhibitor LDN‐193189 reduced cell proliferation of 4T1 cells by over ≈85% over a three‐day culture (Figure S10G, Supporting Information). By contrast, *Bmp7* overexpression caused a ≈ 40% increase in cell proliferation in 4T1 cells (Figure S10H–J, Supporting Information).

When transplanted into the cleared fat‐pad of nude mice, *Bmp7‐*null 4T1 cancer cells grew ≈60% less than control cells as measured by tumor volume (Figure 7I,J). Administration of LDN‐193189 also reduced 4T1 tumor growth by ≈31% (Figure S10K,L, Supporting Information). By contrast, *Bmp7‐*GOF caused a ≈100% increase of 4T1 tumor growth in nude mice (Figure 7K,L).

Together, these data show that, consistent with its role in development, *Bmp7* promotes the progression of triple‐negative breast cancer, and that targeting *Bmp7*, as a part of the interaction loop, is an effective strategy in treating this cancer subtype.

### 2.10 Targeting BMP7‐INHBA Signaling Loop Inhibits Progression of Luminal Breast Cancer Subtype

**Figure 8:**
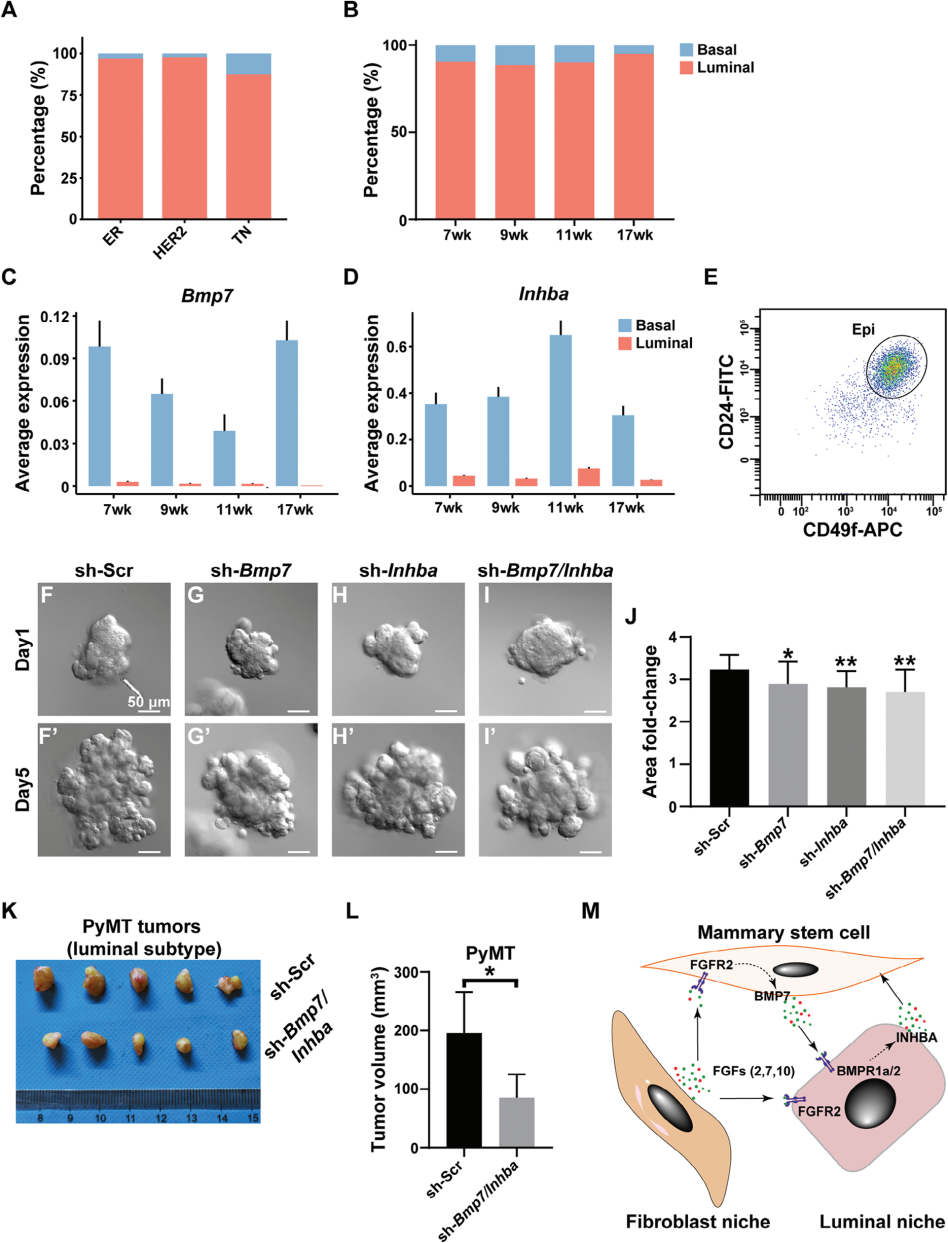
Targeting BMP7‐INHBA signaling loop inhibits progression of luminal breast cancer subtype. A) Percentage distribution of basal and luminal cells in subtypes of human breast cancer. B) Percentage distribution of basal and luminal cells in MMTV‐PyMT tumor at the ages indicated. C,D) Relative mRNA expression of C) *Bmp7* and D) *Inhba* in luminal and basal cells of MMTV‐PyMT mice at the ages indicated. E) Gating strategy for sorting PyMT MECs based on CD24 and Itga6 (CD49f) by FACS. Note that an identical strategy was used to separate luminal cells (CD24^hi^CD49f^low^) from basal cells (CD24^med^CD49f^hi^) (see Figure 1B), but it failed to do so here. F–J) in vitro culture of PyMT MECs transfected with a lentiviral construct expressing either F,F’) a control scrambled shRNA or G,G’) individually sh‐*Bmp7*, H,H’) sh‐*Inhba*, or in combination I,I’) by which *Bmp7* and *Inhba* expression were knocked down using the shRNA technique. MEC outgrowths, as measured by area fold‐change, were quantified (J), (*n* >10). K,L) Tumor growths of PyMT cells 8 weeks post‐surgery in nude mice with reduced expression of *Bmp7* and *Inhba* via shRNA knockdown (K), with tumor volume quantified in (L). Values shown are the mean ± SD for each data point: *P < 0.05; **P < 0.01; ****P < 0.0001. unpaired, two‐tailed Student's *t* tests. M) Schematic diagram of a model depicting the interactions among mammary stem cells and their stromal fibroblastic and luminal epithelial niches. Fibroblast production of FGF ligands, including FGF2, FGF7, and FGF10 are essential for stem cell pool expansion and luminal epithelial morphogenesis. Stromal FGFs are also essential for BMP7 production by MSCs via FGFR2 activation, which activates BMPR1a/2 signaling of luminal niche cells. This in turn upregulates luminal production of INHBA to promote stem cell expansion and completes a positive feedback signaling loop between MSCs and the luminal niche.

Next, we sought to test whether *Bmp7* functions in the luminal cancer subtype. However, we wondered whether there are basal cells, the primary source of BMP7 during development, in the luminal subtype. Therefore, we used published panels of basal and luminal marker genes and analyzed several known scRNA‐seq databases from all three breast cancer subtypes in patients.^[^ ^36^ ^]^ Interestingly, we found luminal cells are by far the most dominant cell type in these cancer subtypes, including in the basal‐like triple‐negative subtype (**Figure** **8**A); however, basal cells are present in all three subtypes as well, including in the luminal subtype (Figure 8A).

Using the scRNA‐seq data from the MMTV‐PyMT mouse line, a model of the luminal cancer subtype,^[^ ^37^, ^38^ ^]^ we confirmed the presence of both basal and luminal cells, with the latter being the predominant cell type (Figure 8B). Moreover, both *Bmp7* and *Inhba* are expressed throughout the cancer progression stages examined (Figure 8C,D). Interestingly, although *Bmp7* is highly expressed in PyMT basal cells as in development, *Inhba* mRNA is not enriched in PyMT luminal cells; rather, it is more enriched in basal cells (Figure 8D), suggesting that gene expression is deregulated in the luminal cancer subtype. Consistent with this notion, we found that the CD24 and CD49f surface markers, traditionally used to separate basal cells from luminal cells, could not effectively do so for the PyMT cancer cells (Figure 8E). Therefore, we decided to examine the consequence of downregulation of both *Bmp7* and *Inhba* expression in PyMT MECs during cancer progression. Using a 3D in vitro PyMT organoid culture, we found that reduced expression of *Bmp7* and/or *Inhba* individually or in combination using the shRNA technique significantly decreased organoid growth (Figure 8F–J). Similarly, using the in vivo transplantation assay, we found that PyMT cells with reduced *Bmp7* and *Inhba* expression had a ≈ 60% reduction in tumor growths (Figure 8K,L).

Together, the data show that a modified form of the BMP7‐INHBA feedback loop is present in different subtypes of breast cancer, and that its blockage inhibits the progression of both the triple‐negative and luminal breast cancer subtypes.

## 3 Discussion

A major challenge in targeted cancer therapy is drug resistance because of cancer heterogeneity. This is partly due to our poor understanding of how subpopulations of cancer cells interact among themselves and with the stromal niche. Here, we demonstrate a reciprocal dependence of the expansion of the basal stem cell pool and luminal epithelium, both of which require input from stromal FGFs during mammary gland regeneration. We show that FGFR2 promotes cell proliferation and BMP7 production in basal stem cells, the latter of which stimulates luminal expansion. Luminal cells form an epithelial niche because they produce INHBA to promote stem cell expansion, thus completing an interaction loop between mammary stem cells and their epithelial and stromal niches (Figure 8M). Finally, the BMP7‐INHBA loop also operates in breast cancer, and targeting one or more components of the loop blocks breast cancer progression. Thus, our study has profound implications for targeting cancer heterogeneity to overcome drug resistance in current cancer therapies.

### 3.1 Luminal Epithelium Is a Mammary Stem Cell Niche

Our results show that stromal FGFs, through their cognate receptor FGFR2, are essential for basal stem cell expansion. This is consistent with the traditional belief that stem cell niches, especially those of vertebrates, are stromal in nature.^[^ ^39^, ^40^, ^41^ ^]^ However, we demonstrate that luminal epithelium, at least in part through INHBA, also promotes the expansion of basal stem cells. Together with previous reports showing that luminal epithelium produces TNF to inhibit the differentiation of basal stem cells,^[^ ^42^ ^]^ the data indicate that luminal epithelium can both positively and negatively regulate mammary stem cell behavior, making it a bona fide stem cell niche.

We show that there is a BMP7‐INHBA positive feedback loop that functions to strengthen mutual dependence and interactions between basal stem cells and their epithelial niche. We and others have previously shown that an FGF‐EGF signaling loop exists between mammary stroma and the epithelium.^[^ ^24^ ^]^ Indeed, such a reciprocal signaling feedback loop is a recurring theme involving two interacting compartments during development. For example, in vertebrate limb development, the interactions between the apical ectodermal ridge, a transient epithelial signaling center, and the underlying mesenchyme are essential for limb bud outgrowth and patterning.^[^ ^43^ ^]^ Together, the data show that interactions between mammary stem cells and their different niches are based on regulatory loops, allowing their reciprocal communication to be readily adjusted depending on the ongoing dynamic changes occurring during organ development and homeostasis.

Finally, it is interesting that *Fgfr2* does not function as a survival factor in either the basal or the luminal epithelium, as judged by the persisted presence and contribution of *Fgfr2* null cells to the epithelial network and, in the case of mutant basal stem cells, their successful differentiation into luminal cells. These results are in stark contrast to many developmental settings, for example, in mid‐/hind‐brain development, FGFR functions as survival factors, and mutant cells undergo apoptosis.^[^ ^44^ ^]^ It is possible that, like in the mammary gland, FGFR is not a survival factor in brain development, and mutant cells may die because of failure to undergo a certain developmental process in the brain. However, a more likely possibility is that FGFR2 plays distinct functions depending on developmental contexts.

### 3.2 Targeting Stem Cell and Niche Interactions as an Effective Cancer Therapy

Several lines of evidence suggest that the FGF‐BMP7‐INHBA signaling loop is essential for mammary gland regeneration and its modified form also operates during breast cancer development, making it a potential target for therapy. First, bioinformatics analysis shows that all breast cancer subtypes have both basal and luminal cells, although the basal‐to‐luminal ratio may vary. This is consistent with a previous report that showed a similar result.^[^ ^45^ ^]^ The main components of the signaling loop, including *Bmp7, Inhba*, and *Fgfr2* are expressed by basal and luminal cells across various stages of cancer development. Finally, compromising the signaling loop, for example, by reducing the expression of one of the above signaling components, leads to decreased cancer growths in vitro and in vivo.

However, there are certain modifications of the FGF‐BMP7‐INHBA signaling loop in the cancer settings. For example, *Inhba* is no longer restricted to luminal cells in the PyMT model, and it is not as strongly dependent on BMP7 stimulation in the 4T1 triple‐negative breast cancer model as in development. These abnormalities could explain why cancer still progresses, though not as rigorously, when the loop has been targeted. Together, our data show that targeting stem cells and niche interactions is effective in treating both triple‐negative breast cancer, in which *Bmp7* is highly expressed (a typical targeting strategy for current therapies), and the PyMT luminal subtype, where *Bmp7* is not overexpressed.

Our results have profound implications for the future of targeted therapy. One of the most significant hurdles of the current targeted therapies is drug resistance, where, no matter how impressive the results of the initial treatments are, cancer cells become resistant, and relapses occur. A main reason, as revealed by recent technological advances in single‐cell biology, is that cancer cells are heterogeneous and current therapies inevitably target only some of them, leading to the eventual buildup of “resistant” sub‐populations. While recognizing the molecular complexity of cancer cells, we focus on targeting stem cell and niche interactions, which we show operate not only in development but also in cancer progression. Our strategy thus deconvolutes the complexity of cancer cell subtypes and target an essential signaling loop for cancer therapy. Future studies should examine whether a similar strategy work in other types of cancers and whether it is effective in human patients with cancer.

## 4 Experimental Section

### Mouse Strains

Immunologically compromised female nude (nu/nu) mice were from Charles River Laboratories. Mice carrying the *R26R* ^mT/mG^ reporter allele (JAX Mice, #007576)^[^ ^30^ ^]^ were purchased from the Jackson Laboratory, while those carrying the *Fgfr2* ^fl^ allele were provided by Dr. David Ornitz. Offspring from crosses of the various lines were genotyped according to methods in the publications describing the mouse lines. The mice were housed and maintained in accordance with regulations from South China University's Institutional Animal Care and Use Committee.

### Preparation of Mammary Gland Epithelial Cells

Extraction of primary mammary gland organoids was performed as previously described.^[^ ^46^ ^]^ Briefly, mouse # 2, 3, 4, and 5 mammary glands were collected in 10 mL collagenase solution (DMEM/F12, containing 2 mg mL^−1^ collagenase (Sigma, C5138), 2 mg mL^−1^ Trypsin (Gibco, 27250018), 5 µg mL^−1^ insulin (Yeasen, 40107ES25), 5% fetal bovine serum (FBS) (Gibco, 10099141), 50 µg mL^−1^ Gentamicin), in which they were digested for 30 min. Mammary organoids were pelleted by centrifugation at 450 *g* for 10 min and then purified by 3–5 rounds of differential centrifugations. Mammary gland organoids were digested in a 0.25% Trypsin /EDTA (Meilunbio, MA0233) water bath for 12 min into single cells for subsequent sorting or virus infection.

### Construct Production and Virus Infection

Design of shRNA sequence was based on BLOCK‐iT RNAi Designer provided by Thermo Fisher Scientific. The pLKO.1 (Addgene# 8453) vector was digested with AgeI‐HF and EcoRI‐HF, then ligated with annealed shRNA fragment using the T4 DNA ligase (Abclonal, RK21501), and validated by Sanger sequencing. Knockdown efficiency was evaluated using the HC11 cells or basal cells, and the constructs with the highest efficiency were selected for subsequent experiments. For construction of overexpression vectors, *Bmp7*, *Bmpr1a*, *Bmpr2* full‐length CDS regions were cloned into pLeGO‐SFFV‐P2A‐mCherry using the ClonExpress Ultra One Step Cloning Kit (Vazyme Biotech co., ltd., C115). Plasmids were prepared using the endotoxin‐free plasmid extraction kit (Magen, P1112).

The sequences of shRNAs were as follows:

Scramble shRNA: 5′‐CCTAAGGTTAAGTCGCCCTCG‐3′;
*Bmp7*‐shRNA: 5′‐GGATCTATAAGGACTACATCC‐3′;
*Bmpr1a*‐shRNA: 5′‐GGAGAAACCACATTAACTTCT‐3′;
*Bmpr2*‐shRNA: 5′‐GCCAAGATGAATACAATCAAT‐3′;
*Inhba*‐shRNA: 5′‐CCTTCCACTCAACAGTCATTAT‐3′;

Briefly, HEK293T cells were cotransfected with pMD.2, pspax2, and transfer plasmids, using the Eztrans (Shanghai Life‐iLab Biotech Co., Ltd, AC04L099). The medium containing the virus was harvested twice at 60 and 88 h after transfection. Centrifuge at 27 000 rpm for 2 h to concentrate the lentivirus. Resuspend the lentivirus in DMEM/F12 containing 10% FBS and store at −80 °C.

For viral infection, freshly extracted mammary gland organoids or cell line cells were cultured in primary mammary gland culture medium (DMEM/F12, containing 10% FBS, 10 ng mL^−1^ EGF, 5 µg mL^−1^ insulin, 1 µg mL^−1^ Hydrocortisone, 1% L‐Ala‐Gln, and 1% penicillin and streptomycin(P/S)). Infection was done overnight for adenovirus, or 2 h for lentivirus in an ultra‐low attachment culture dish. Flow sorting was carried out to get fluorescent protein positive cells.

### Fluorescence‐Activated Cell Sorting (FACS)

Primary mammary gland cells or other cells were dissociated and were fluorescently labeled by infection using Ad‐Cre‐GFP virus, lentivirus, or antibody staining, and separated by DAPI or Fixable Viability Dye eFluor 780 (Invitrogen, 65‐0865‐14) to choose live cells. Antibodies against CD24‐eFluor450 (Invitrogen, 48‐0242‐82) and CD49f‐APC (Invitrogen, 17‐0495‐82) were used to sort for luminal and basal populations. Sorting was done using an AriaIII system and data were processed using FlowJo10 software.

### Colony‐Forming Assay

500 or 300 basal or luminal cells were cultured in ultra‐low attachment 96‐well plates, the medium was stem cell medium, advanced DMEM/F12 containing 5% FBS, 10 ng mL^−1^ EGF, 20 ng mL^−1^ FGF2, 4 µg mL^−1^ heparin, 5 µM Y‐27632, 1% L‐Alu‐Gln, 1% P/S and 5% Matrigel. After 7 d of culture, the statistic is greater than 50 µm number of clones. For medium in which BMP7 (R&D Systems, 5666‐BP) or INHBA (Novoprotein, C687A) was used, their respective concentration was 50  or 5 ng mL^−1^. To prepare conditioned medium, 100 000 basal or luminal cells were cultured in 1 mL of medium for 2 d, the medium was collected and passed through a 0.45 µm filter, and then frozen at ‐20 °C. After that, 5% Matrigel was added again and the cells were seeded for experiments.

### Quantitative Real‐Time PCR

RNA extraction kit (Magen, R4012) was used to prepare total RNA from cells. Equal amounts of RNA templates were reverse transcribed into cDNAs using HiScript III RT SuperMix for qPCR (Vazyme Biotech Co., Ltd, R323). Then cDNAs were used in qPCR reactions using ChamQ Universal SYBR qPCR Master Mix (Vazyme Biotech co., ltd, Q711) with the BIO‐RAD CFX Connect Real‐Time Systems according to the manufacturer's protocol. *Actb* served as an internal control. Primer sequences are listed in Table S1 (Supporting Information).

### Coculture of Basal and Luminal Cells

1000 basal or luminal cells were harvested and put into a well of a 96‐well round bottom ultralow attachment plate to aggregate either alone or as a mixture (1:1 ratio) overnight in a 37 °C oven. Aggregates were then cultured in an eight‐well chamber slide in basic medium containing DMEM/F12 with 1xITS, 1% P/S, and 10 ng mL^−1^ FGF2 (GenScript, Z03116). Cultures were imaged under a Zeiss Observer Z1 microscope. Areas and branches were analyzed using ImageJ for statistics.

### Growth Factor Stimulation

After the cells were cultured as spheres in the stem cell medium, they were placed in a sphere containing 2% Growth factor, starved for 24 h in the basic medium of reduced Matrigel, treated with FGF2, FGF7 (GenScript, Z03154), or FGF10 (GenScript, Z03155) (50 ng mL^−1^) or BMP7 (50 ng mL^−1^) for 24 h, before RNA or proteins were extracted for downstream experiments. For HC11, RPMI 1640 containing, 1% L‐Alu‐Gln, and 1% P/S were starved for 16 h, then stimulated with BMP7 (50 ng mL^−1^) for 30 min or 6 h, and RNA or protein was extracted for downstream experiments.

### Mammary Gland Transplantation and Visualization

For mammary gland regeneration transplantation, 3‐week‐old female nude mice were used, and a total of 20 000 mammary gland epithelial cells were injected into cleared fat‐pad, mammary glands were collected after waiting 2–8 weeks. The mammary glands were tiled on the adhesive glass slides, and directly used for fluorescence imaging with Zwiss Axio Zoom V16 stereoscope; or treated with Carnoy ’s fixative solution, rehydrated, treated with carmine staining solution, dehydrated, and degreased before imaging. For the breast tumor orthotopic transplantation experiment, 6‐week‐old nude mice were used and injected 100 000 4T1 or PyMT cells into the fat pad without removing the mammary gland tissue in situ. For 4T1 cells, 3 weeks were waited to harvest the tumors, and for PyMT cells, 8 weeks were waited, ensuring that the tumor size at collection did not exceed animal welfare requirements (i.e., no dimension exceeding 1 cm). Subsequently, tumor mass was measured using a balance, and tumor volume was measured using calipers. The volume calculation formula is volume = length × width^2 × 0.5. LDN‐193189 (3 mg kg^−1^ body weight) was administered by intraperitoneal injection once every 2 d.

### Immunofluorescence Analysis

Mammary glands were harvested and fixed in 4% paraformaldehyde overnight at 4 °C. Then they were soaked in 15% sucrose and 30% sucrose prepared in PBS for 12 h. 10 µm frozen sections were cut using a Leica cryostat. Sections were permeabilized with 0.5% Triton X‐100 in PBS for 45 minutes. Sections were blocked for 2 h at room temperature (RT) in PBS containing 10% goat serum and 0.2% Tween‐20, followed by incubation in primary antibodies overnight at 4 °C. Incubation with secondary antibodies for 2 h at 25 °C. DAPI Nuclei were visualized by DAPI staining. Confocal microscopy was performed on a Zeiss LSM 800 Confocal. Primary antibodies used in this study were K8 antibody (DSHB, #TROMA‐I, 1:500 dilution), Ki67 antibody (Abcam, #ab16667, 1:200 dilution).

### Culture of Cell Lines and Proliferation Assays

HEK293T and 4T1 cells were cultured in DMEM (Gibco, # C12430500BT), supplemented with 10% FBS (Lonsera, S711‐001S), 1% sodium pyruvate, 1% nonessential amino acid, 1% L‐Ala‐Gln, and 1% P/S. HC11 cells were cultured in RPMI 1640 (Gibco, #C11875500CP), supplemented with 10% FBS, 10 ng mL^−1^ EGF, 5 µg mL^−1^ insulin, 1% L‐Ala‐Gln, and 1% P/S. Cells were tested by PCR and confirmed to be free of mycoplasma.

For CCK8 assay, cells were cultured in a 96‐well plate for the indicated durations. They were then cultured with the fresh medium containing 10% Cell Counting Kit‐8 (CCK8, Share‐Bio, SB‐CCK8) for 2 h before absorbance at 450 nm was taken using a microplate reader. For Edu labeling assays, tumor cells were cultured in a 96‐well plate for 36 h, 10 µM EdU solution was added and incubated for 1 h. Afterward, staining was performed according to the instructions of the EdU Cell Proliferation Kit (Epizyme, CX003), and Nikon TIRF Microscope was used for imaging, and Image J was used for data statistics.

### Construction of CRISPR‐Mediated Knockout Cell Line

CRISPR/Cas9 technique was used for the generation of *Bmp7* knock‐out 4T1 cell line. Briefly, using Smart‐CRISPR (https://www.cyagen.com/cn/zh‐cn/cellbank‐out/gene.html) to design sgRNA and connect it to LentiCRISPR.v2 (Addgene, #52961) vector. The plasmid was transfected into 4T1 cells using lipofection regent (Yeasen, 40802ES02), and after 48 h, the cells were sorted into 96‐well plates by flow cytometry to construct monoclonal cell lines. Finally, use sanger sequencing and western blotting to determine the knockout of the single‐cell cloning.

### Western Blotting Assays

HC11 cell preparations were lysed in buffer containing 20 × 10^−3^ m Tris‐HCl (pH7.4), 150 × 10^−3^ m NaCl, 1 mM EDTA, 1 mM EGTA, 5 mM NaF, 1 mM orthovanadate, 10% (vol/vol) glycerol, 1% Triton X‐100, 0.5% Nonidet P‐40, 1 × 10^−3^ m phenylmethylsulfonyl fluoride, 2 µg mL^−1^ leupeptin, and 10 µg mL^−1^ aprotinin. Samples were cleared with centrifugation at 13500 x *g* at 4 °C for 10 min. Protein concentrations were pre‐quantified with a Pierce BCA assay kit (Thermo Fisher Scientific), and 5–10 µg was resolved by SDS/PAGE under reducing conditions in regular Tris‐glycine buffer. Proteins were electrically transferred in a wet‐ tank to a PVDF membrane. After blocking with 5% BSA (sigma#WXBC3116V), target proteins were visualized using anti‐SMAD1(Cell Signaling

Technology (CST), #6944S, 1:1000), anti‐SMAD5(CST, #12534S, 1:1000), anti‐phospho‐SMAD1/5(CST, #9516S, 1:1000), anti‐BMP7(Santa Cruz Biotech, #sc‐53917, 1:1000), anti‐INHBA (ABclonal, #A5232, 1:1000), anti‐GAPDH (ABclonal, #AC033, 1:100000). After the primary antibody was incubated and extensively washed, the membrane was reacted with the HRP‐conjugated secondary antibody at room temperature for 2 h. The reactive bands were developed by ECL kit (EpiZyme, SQ202) and tested with Amersham Imager 680. For the quantification of protein expression, band density was measured using Image J software.

### Construction of cDNA Libraries and bulk RNA Sequencing

1 µg total RNA was used for the following library preparation. The poly(A) mRNA isolation was performed using Oligo(dT) beads. The mRNA fragmentation was performed using divalent cations and high temperatures. Priming was performed using Random Primers. First‐strand cDNA and the second‐strand cDNA were synthesized. The purified double‐stranded cDNA was then treated to repair both ends and add a dA‐tailing in one reaction, followed by a T–A ligation to add adaptors to both ends. Size selection of Adaptor‐ligated DNA was then performed using DNA Clean Beads. Each sample was then amplified by PCR using P5 and P7 primers and the PCR products were validated.

Then libraries with different indexes were multiplexed and loaded on an Illumina Novaseq 6000 instrument for sequencing using a 2×150 paired‐end (PE) configuration according to manufacturer's instructions. The library construction and sequencing were performed by GENEWIZ (Suzhou, China).

Raw reads in fastq format were aligned to mouse genome mm10 using subjunc function in subread package (Version 2.0.3). Mapped reads for each sample were counted for each gene in annotation files in GTF format (genecode.vM30.annotation.gtf), using the FeatureCounts function in subread package. Differential gene analysis was performed by DESeq2 R package, while clusterProfiler R package was used to GSEA analysis. Database of ligand–receptor interaction was downloaded from (github.com/arc85/celltalker) using the celltalker R package.

### Single Cell RNA Sequencing and Bioinformatics Analysis

The Cell Ranger V2.0.2 tool was used for quality control and quantification of the original sequencing data from 10X genomics. The aggr function in Cell Ranger was used to integrate data from control and experimental samples. The Seurate V3 R package was used for data input, quality control, clustering, and differential gene expression analysis.^[^ ^32^ ^]^ CD45 was used to filter out immune cells, leaving 157 cells in the control sample, while 33 cells in the experimental sample. Published marker gene sets were used to differentiate basal and luminal cells.^[^ ^33^ ^]^ The top 500 most differentially expressed genes were used, of which 46 were chosen as marker genes for basal and luminal cell analysis. The CellphoneDB app was used to analyze genes involved in cell‐cell communication,^[^ ^34^ ^]^ while the ClusterProfiler R kit was used for GSEA analysis.^[^ ^47^ ^]^ Differentially expressed genes were further validated using Wilcox software.

### Bioinformatics Analysis of BMP‐BMPR, FGF‐FGFR mRNA Expression

The scRNA‐seq data on different stages of mammary gland development, different stages of MMTV‐PyMT mouse breast cancer, and different hormone receptor subtype of human breast cancer were obtained from GSE164017,^[^ ^28^ ^]^ PRJNA762594 and GSE161529, respectively. Data input, quality control, integration, and cell clustering were performed by Seurat R package.^[^ ^32^ ^]^ *Krt14* and *Krt5* were used to mark basal cells, where *Krt18* and *Krt8* luminal cells. Gene expression was then averaged across different stages in basal and luminal cells.

### Analysis of BMP7/INHBA mRNA Expression in Breast Cancer and Survival Probability

Data on *BMP7*/*INHBA* mRNA expression in breast cancer subtypes were based on The Cancer Genome Atlas (TCGA) Breast Cancer (BRCA) datasets, available at UCLAN (http://ualcan.path.uab.edu/analysis.html). Survival probability analysis was based on data on 534 breast cancer patients. Kaplan‐Meier Plotter (http://kmplot.com/analysis/index.php?p = service&cancer = breast) was used for the analysis.

### Statistics

Sample size for each figure is denoted in the figure legends. Statistical significance between conditions was assessed by two‐tailed Student's t‐tests. All error bars represent SD, and significance is denoted as *P < 0.05, **P < 0.01, ***P < 0.001 and ****P < 0.0001. n.s. denotes not significant.

### Data Availability

The bulk RNA sequencing and single‐cell RNA sequencing data were deposited in NCBI's Gene Expression Omnibus (GEO) repository and are accessible through GEO Series accession numbers GSE224902 and GSE224418, respectively. All data supporting the conclusions of this study are provided in the main text or Supporting Information.

## Conflict of Interest

The authors declare no conflict interests.

## Author Contributions

R.M., D.F., and J.C. contributed equally to this work. R.M., transplantation experiments and analyses, functional assays for *Bmp7*, *Bmpr1a/2*, *Inhba*, and *Fgfr2*, cancer studies; D.F., scRNA‐seq, bioinformatic analysis, development of basal and luminal coculture assays; J.C., confocal imaging, cancer studies; J.Z, K.X., X.K., and G.H., review and editing; P.L., conceptualization, funding acquisition, data curation, and writing.

## Supporting information

Supporting Information

Supporting Information

## Data Availability

The data that support the findings of this study are available from the corresponding author upon reasonable request.

## References

[1] D. J. Konieczkowski , C. M. Johannessen , L. A. Garraway , Cancer Cell 2018, 33, 801. 29763622 10.1016/j.ccell.2018.03.025 PMC5957297

[2] C. J. Watson , FEBS J. 2021, 288, 6082. 34719877 10.1111/febs.16228

[3] L. A. Garraway , E. S. Lander , Cell 2013, 153, 17. 23540688 10.1016/j.cell.2013.03.002

[4] N. McGranahan , C. Swanton , Cell 2017, 168, 613. 28187284 10.1016/j.cell.2017.01.018

[5] D. A. Lawson , N. R. Bhakta , K. Kessenbrock , K. D. Prummel , Y. Yu , K. Takai , A. Zhou , H. Eyob , S. Balakrishnan , C.‐Y. Wang , P. Yaswen , A. Goga , Z. Werb , Nature 2015, 526, 131. 26416748 10.1038/nature15260 PMC4648562

[6] N. Vasan , J. Baselga , D. M. Hyman , Nature 2019, 575, 299. 31723286 10.1038/s41586-019-1730-1 PMC8008476

[7] D. A. Lawson , K. Kessenbrock , R. T. Davis , N. Pervolarakis , Z. Werb , Nat. Cell Biol. 2018, 20, 1349. 30482943 10.1038/s41556-018-0236-7 PMC6477686

[8] R. B. Roberts , C. L. Arteaga , D. W. Threadgill , Cancer Cell 2004, 5, 115. 14998487 10.1016/s1535-6108(04)00032-7

[9] I. Martincorena , A. Roshan , M. Gerstung , P. Ellis , P. Van Loo , S. McLaren , D. C. Wedge , A. Fullam , L. B. Alexandrov , J. M. Tubio , L. Stebbings , A. Menzies , S. Widaa , M. R. Stratton , P. H. Jones , P. J. Campbell , Science 2015, 348, 880. 25999502 10.1126/science.aaa6806 PMC4471149

[10] C. Tomasetti , B. Vogelstein , G. Parmigiani , Proc. Natl. Acad. Sci. USA 2013, 110, 1999. 23345422 10.1073/pnas.1221068110 PMC3568331

[11] A. Sreekumar , K. Roarty , J. M. Rosen , Endocr.‐Relat. Cancer 2015, 22, T161. 26206777 10.1530/ERC-15-0263 PMC4618079

[12] G. Sflomos , K. Schipper , T. Koorman , A. Fitzpatrick , S. Oesterreich , A. V. Lee , J. Jonkers , V. G. Brunton , M. Christgen , C. Isacke , P. W. B. Derksen , C. Brisken , Cancers 2021, 13, 5396. 34771558 10.3390/cancers13215396 PMC8582475

[13] R. Eyre , D. G. Alférez , A. Santiago‐Gómez , K. Spence , J. C. McConnell , C. Hart , B. M. Simões , D. Lefley , C. Tulotta , J. Storer , A. Gurney , N. Clarke , M. Brown , S. J. Howell , A. H. Sims , G. Farnie , P. D. Ottewell , R. B. Clarke , Nat. Commun. 2019, 10, 5016. 31676788 10.1038/s41467-019-12807-0 PMC6825219

[14] I. S. Kim , Y. Gao , T. Welte , H. Wang , J. Liu , M. Janghorban , K. Sheng , Y. Niu , A. Goldstein , N. Zhao , I. Bado , H.‐C. Lo , M. J. Toneff , T. Nguyen , W. Bu , W. Jiang , J. Arnold , F. Gu , J. He , D. Jebakumar , K. Walker , Y. Li , Q. Mo , T. F. Westbrook , C. Zong , A. Rao , A. Sreekumar , J. M. Rosen , X. H.‐F. Zhang , Nat. Cell Biol. 2019, 21, 1113. 31451770 10.1038/s41556-019-0373-7 PMC6726554

[15] J. E. Visvader , J. Stingl , Genes Dev. 2014, 28, 1143. 24888586 10.1101/gad.242511.114 PMC4052761

[16] A. Van Keymeulen , A. S. Rocha , M. Ousset , B. Beck , G. Bouvencourt , J. Rock , N. Sharma , S. Dekoninck , C. Blanpain , Nature 2011, 479, 189. 21983963 10.1038/nature10573

[17] M. Shackleton , F. Vaillant , K. J. Simpson , J. Stingl , G. K. Smyth , M.‐L. Asselin‐Labat , L. Wu , G. J. Lindeman , J. E. Visvader , Nature 2006, 439, 84. 16397499 10.1038/nature04372

[18] J. Stingl , P. Eirew , I. Ricketson , M. Shackleton , F. Vaillant , D. Choi , H. I. Li , C. J. Eaves , Nature 2006, 439, 993. 16395311 10.1038/nature04496

[19] B. A. Howard , P. Lu , Semin. Cell Dev. Biol. 2014, 25–26, 43. 10.1016/j.semcdb.2014.01.004 24445189

[20] P. Lu , V. M. Weaver , Z. Werb , J. Cell Biol. 2012, 196, 395. 22351925 10.1083/jcb.201102147 PMC3283993

[21] E. Sahai , I. Astsaturov , E. Cukierman , D. G. DeNardo , M. Egeblad , R. M. Evans , D. Fearon , F. R. Greten , S. R. Hingorani , T. Hunter , R. O. Hynes , R. K. Jain , T. Janowitz , C. Jorgensen , A. C. Kimmelman , M. G. Kolonin , R. G. Maki , R. S Powers , E. Puré , D. C. Ramirez , R. Scherz‐Shouval , M. H. Sherman , S. Stewart , T. D. Tlsty , D. A. Tuveson , F. M. Watt , V. Weaver , A. T. Weeraratna , Z. Werb , Nat. Rev. Cancer 2020, 20, 174. 31980749 10.1038/s41568-019-0238-1 PMC7046529

[22] S. Attaran , M. J. Bissell , Semin. Cancer Biol. 2022, 78, 35. 34757184 10.1016/j.semcancer.2021.09.008 PMC9605861

[23] P. Lu , T. Zhou , C. Xu , Y. Lu , Wiley Interdiscip. Rev. Dev. Biol. 2019, 8, e357. 31322329 10.1002/wdev.357

[24] Z. Koledova , X. Zhang , C. Streuli , R. B. Clarke , O. D. Klein , Z. Werb , P. Lu , Proc. Natl. Acad. Sci. USA 2016, 113, E5731. 27621461 10.1073/pnas.1611532113 PMC5047180

[25] X. Zhang , D. Martinez , Z. Koledova , G. Qiao , C. H. Streuli , P. Lu , Development 2014, 141, 3352. 25078648 10.1242/dev.106732 PMC4199126

[26] O. K. Sirka , E. R. Shamir , A. J. Ewald , J. Cell Biol. 2018, 217, 3368. 30061105 10.1083/jcb.201802144 PMC6168248

[27] Y. Lu , R. Deng , H. You , Y. Xu , C. Antos , J. Sun , O. D. Klein , P. Lu , Cell Rep. 2020, 33, 108246. 33053348 10.1016/j.celrep.2020.108246 PMC7668195

[28] B. Pal , Y. Chen , M. J. G. Milevskiy , F. Vaillant , L. Prokopuk , C. A. Dawson , B. D. Capaldo , X. Song , F. Jackling , P. Timpson , G. J. Lindeman , G. K. Smyth , J. E. Visvader , Breast Cancer Res. 2021, 23, 69. 34187545 10.1186/s13058-021-01445-4 PMC8243869

[29] D. M. Ornitz , J. Xu , J. S. Colvin , D. G. McEwen , C. A. MacArthur , F. Coulier , G. Gao , M. Goldfarb , J. Biol. Chem. 1996, 271, 15292. 8663044 10.1074/jbc.271.25.15292

[30] M. Muzumdar , B. Tasic , K. Miyamichi , L. Li , L. J. g. Luo , Genesis 2007, 45, 593. 17868096 10.1002/dvg.20335

[31] P. Lu , A. J. Ewald , G. R. Martin , Z. Werb , Dev. Biol. 2008, 321, 77. 18585375 10.1016/j.ydbio.2008.06.005 PMC2582391

[32] T. Stuart , A. Butler , P. Hoffman , C. Hafemeister , E. Papalexi , W. M. Mauck 3rd , Y. Hao , M. Stoeckius , P. Smibert , R. Satija , Cell 2019, 177, 1888. 31178118 10.1016/j.cell.2019.05.031 PMC6687398

[33] B. Pal , Y. Chen , F. Vaillant , P. Jamieson , L. Gordon , A. C. Rios , S. Wilcox , N. Fu , K. H. Liu , F. C. Jackling , M. J. Davis , G. J. Lindeman , G. K. Smyth , J. E. Visvader , Nat. Commun. 2017, 8, 1627. 29158510 10.1038/s41467-017-01560-x PMC5696379

[34] M. Efremova , M. Vento‐Tormo , S. A. Teichmann , R. Vento‐Tormo , Nat. Protoc. 2020, 15, 1484. 32103204 10.1038/s41596-020-0292-x

[35] B. Schrörs , S. Boegel , C. Albrecht , T. Bukur , V. Bukur , C. Holtsträter , C. Ritzel , K. Manninen , A. D. Tadmor , M. Vormehr , U. Sahin , M. Löwer , Front. Oncol. 2020, 10, 1195. 32793490 10.3389/fonc.2020.01195 PMC7390911

[36] B. Pal , Y. Chen , F. Vaillant , B. D. Capaldo , R. Joyce , X. Song , V. L. Bryant , J. S. Penington , L. Di Stefano , N. Tubau Ribera , S. Wilcox , G. B. Mann , A. T. Papenfuss , G. J. Lindeman , G. K. Smyth , J. E. Visvader , EMBO J. 2021, 40, 107333. 10.15252/embj.2020107333 PMC8167363 33950524

[37] G. Jiang , J. Tu , L. Zhou , M. Dong , J. Fan , Z. Chang , L. Zhang , X. Bian , S. Liu , Cell Death Dis. 2021, 12, 979. 34675206 10.1038/s41419-021-04261-y PMC8531288

[38] S. Attalla , T. Taifour , T. Bui , W. Muller , Oncogene 2021, 40, 475. 33235291 10.1038/s41388-020-01560-0 PMC7819848

[39] N. McCarthy , J. Kraiczy , R. A. Shivdasani , Nat. Cell Biol. 2020, 22, 1033. 32884148 10.1038/s41556-020-0567-z

[40] K. A. U. Gonzales , E. Fuchs , Dev. Cell 2017, 43, 387. 29161590 10.1016/j.devcel.2017.10.001 PMC5797699

[41] K. J. Roberts , A. M. Kershner , P. A. Beachy , Cancer Cell 2017, 32, 404. 29017054 10.1016/j.ccell.2017.08.007 PMC5679442

[42] A. Centonze , S. Lin , E. Tika , A. Sifrim , M. Fioramonti , M. Malfait , Y. Song , A. Wuidart , J. Van Herck , A. Dannau , G. Bouvencourt , C. Dubois , N. Dedoncker , A. Sahay , V. de Maertelaer , C. W. Siebel , A. Van Keymeulen , T. Voet , C. Blanpain , Nature 2020, 584, 608. 32848220 10.1038/s41586-020-2632-y PMC7116172

[43] P. Lu , Y. Yu , Y. Perdue , Z. Werb , Development 2008, 135, 1395. 18359901 10.1242/dev.018945 PMC2574509

[44] C. L. Chi , S. Martinez , W. Wurst , G. R. Martin , Development 2003, 130, 2633. 12736208 10.1242/dev.00487

[45] K. J. Cheung , E. Gabrielson , Z. Werb , A. J. Ewald , Cell 2013, 155, 1639. 24332913 10.1016/j.cell.2013.11.029 PMC3941206

[46] Y. Lu , R. Deng , H. You , P. Lu , STAR Protoc. 2021, 2, 100778. 34485944 10.1016/j.xpro.2021.100778 PMC8405933

[47] G. Yu , L. G. Wang , Y. Han , Q.‐Y. He , OMICS 2012, 16, 284. 22455463 10.1089/omi.2011.0118 PMC3339379

[48] K. Bach , S. Pensa , M. Grzelak , J. Hadfield , D. J. Adams , J. C. Marioni , W. T. Khaled , Nat. Commun. 2017, 8, 2128. 29225342 10.1038/s41467-017-02001-5 PMC5723634

