# Supplementary material for: Targeting Tumor Heterogeneity by Breaking a Stem Cell and Epithelial Niche Interaction Loop
Source: Adv Sci (Weinh). 2024 May 6;11(26):2307452. doi: 10.1002/advs.202307452 (PMC11234407; doi:10.1002/advs.202307452)
Supplement: Supplementary file 1 — Supporting Information [file ADVS-11-2307452-s001.pdf]

ADVANCED SCIENCE
Open Access

Supporting Information

for *Adv. Sci.*, DOI 10.1002/advs.202307452

Targeting Tumor Heterogeneity by Breaking a Stem Cell and Epithelial Niche Interaction Loop

*Rongze Ma, Deyi Feng, Jing Chen, Jiecan Zhou, Kun Xia, Xiangyin Kong, Guohong Hu and Pengfei Lu**

SUPPLEMENTARY FIGURE LEGENDS

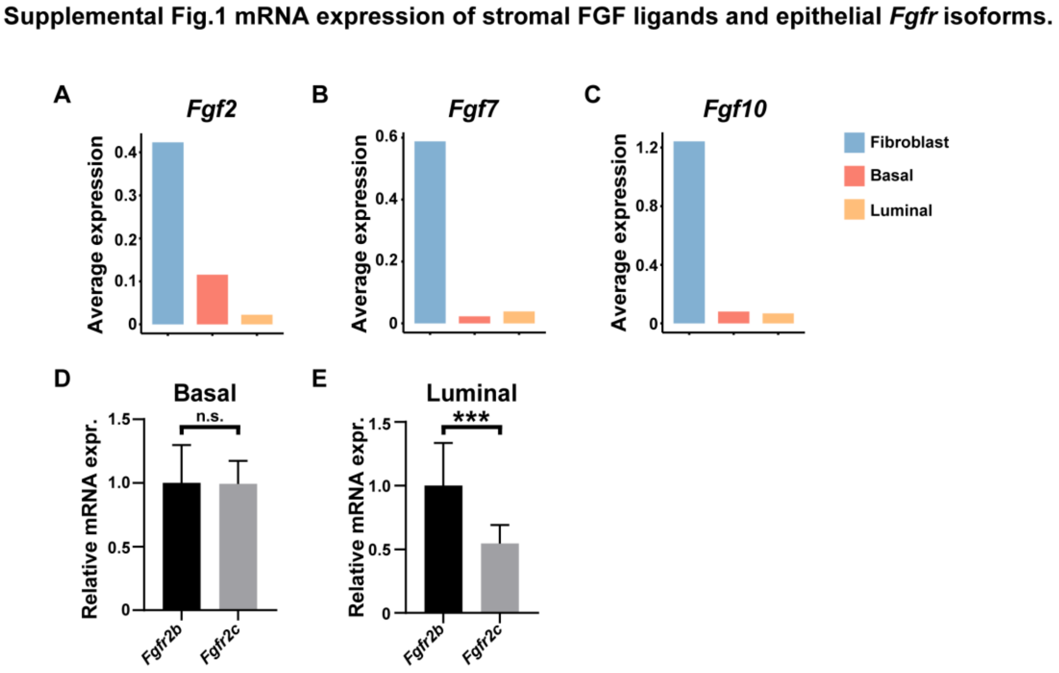

Supplementary Figure S1: mRNA expression of stromal FGF ligands and epithelial *Fgfr*

isoforms.

(**A**-**C**) mRNA expression of *Fgf2* (**A**), *Fgf7* (**B**), and *Fgf10* (**C**)in the developing mammary gland at the seven-week stage based on scRNA-seq datasets.

(**D**-**E**) Relative expression of the b and c isoforms of *Fgfr2* in sorted basal (**D**) and luminal (**E**) cells. Data were mean ± SD; n.s., not significant, $P \geq 0.05$; *** $P < 0.001$.

Supplymental Fig. 2. ***Fgfr2*** **promotes luminal epithelial expansion during mammary gland regeneration.**

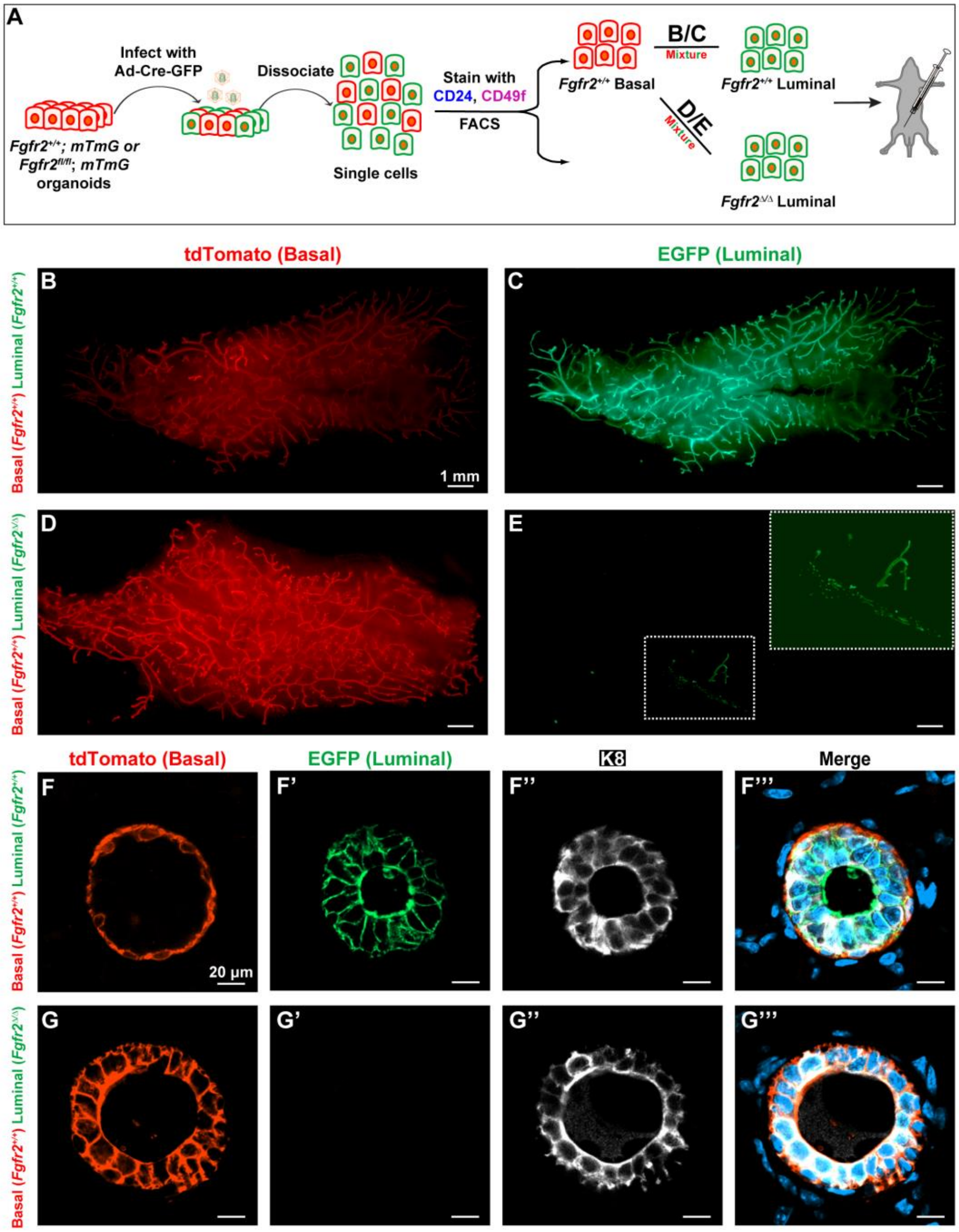

Supplementary Figure S2: ***Fgfr2*** **promotes luminal epithelial branching and expansion**

during mammary gland regeneration.

(**A**) Schematic diagram depicting the experimental procedure of sample preparation, adenoviral infection, FACS, and transplantation strategy. ~ 20,000 cells were injected into the cleared fat pad.

(**B-G'''**) Wholemount (**B-E**) or immunofluorescence on frozen sections (**F-G'''**) of the epithelial network 8 weeks after transplantation. The tdTomato channel allowed visualization of red wild-type basal cells (**B**, **D**); the EGFP channel allowed visualization of epithelial cells derived from green wild-type (**C**) and *Fgfr2*$^{\Delta/\Delta}$ null (**E**) luminal cells. Note that the tdTomato fluorescence was much brighter in the experimental gland (**D**) than in the control gland (**B**). Also note that K8^{+} luminal cells were green in the control gland (**F-F'''**), but were red in the experimental gland (**G-G'''**).

Supplymental Fig. 3. *Fgfr2* promotes luminal epithelial expansion during mammary gland regeneration.

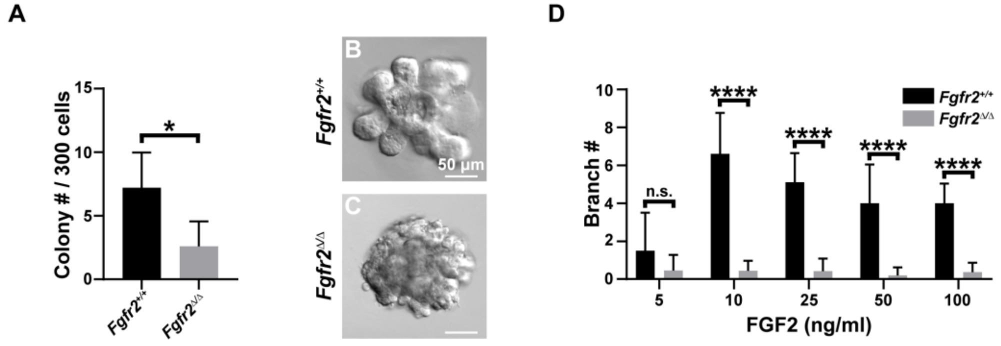

Supplementary Figure S3: *Fgfr2* promotes luminal epithelial expansion during mammary

gland regeneration.

(**A**) Quantitative analysis of colony-forming efficiency by wild-type and *Fgfr2*$^{\Delta/\Delta}$ null luminal cells.

(**B**-**D**) In vitro branching assay in which purified wild-type (**B**) and *Fgfr2*$^{\Delta/\Delta}$ null (**C**) luminal cells were cultured in basal medium containing a progressively higher concentration of FGF2. Scale bars: 100 μm. Branch number was quantified at each FGF2 concentration (**D**).

Values shown are the mean ± SD (n > 10) for each data point. Unpaired Student's t-test was performed for statistical analysis, n.s., not significant, $P \geq 0.05$; * $P < 0.05$; **** $P < 0.0001$.

Supplemental Fig.4. FGFR2 signaling promotes *Bmp7* expression in basal cells.

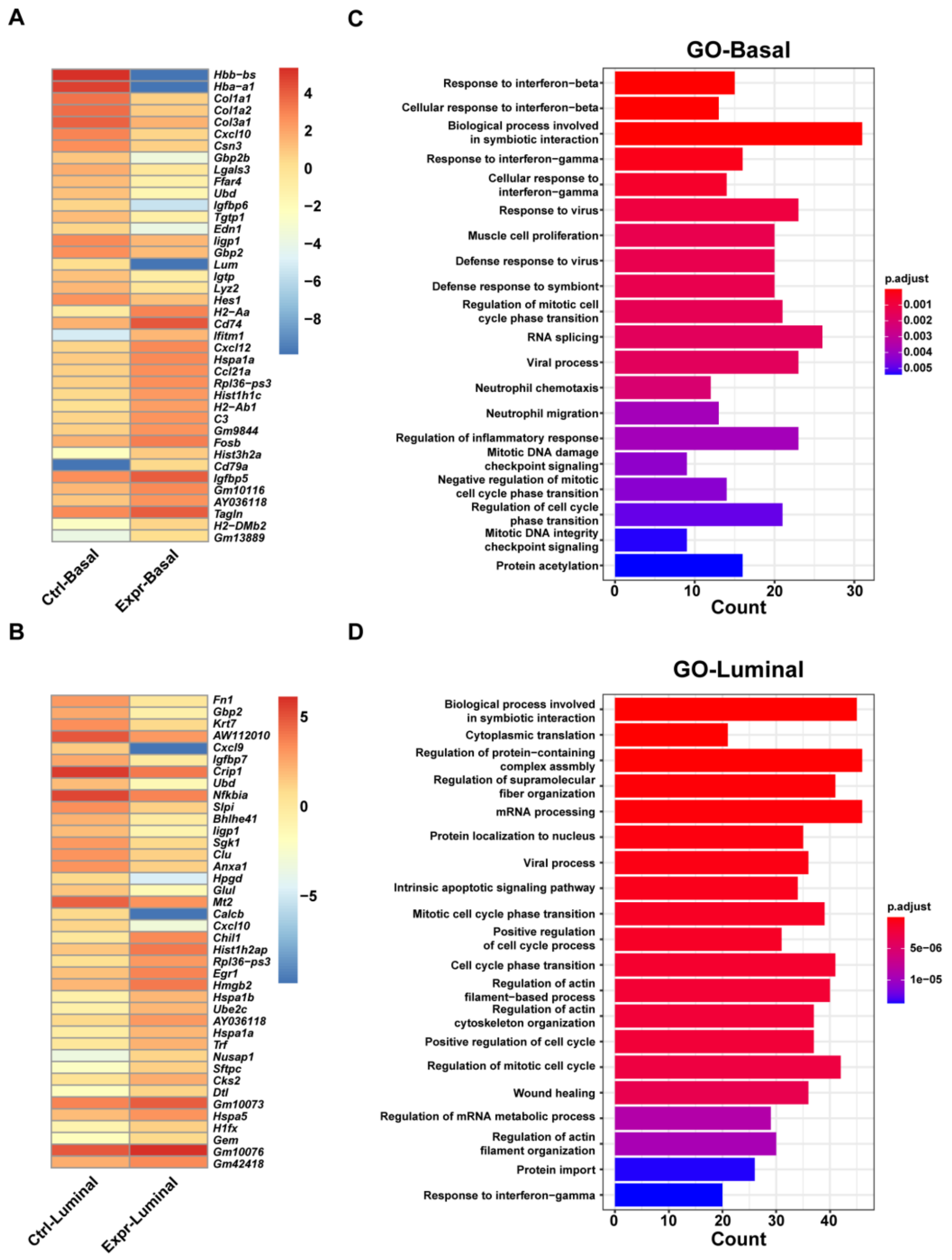

Supplementary Figure S4: Differentially expressed genes and changes in cellular pathways

in basal and luminal cells as a result of *Fgfr2* loss in basal cells.

(**A**, **B**) Top 40 differentially expressed genes in basal cells (**A**) and luminal cells (**B**) of control and experimental transplants, derived from wild-type luminal cells mixed with wild-type basal cells or *Fgfr2*$^{\Delta/\Delta}$ basal cells, respectively. (**C**, **D**) GO analysis of main pathway changes in basal cells (**C**) and luminal cells (**D**) of control and experimental transplants.

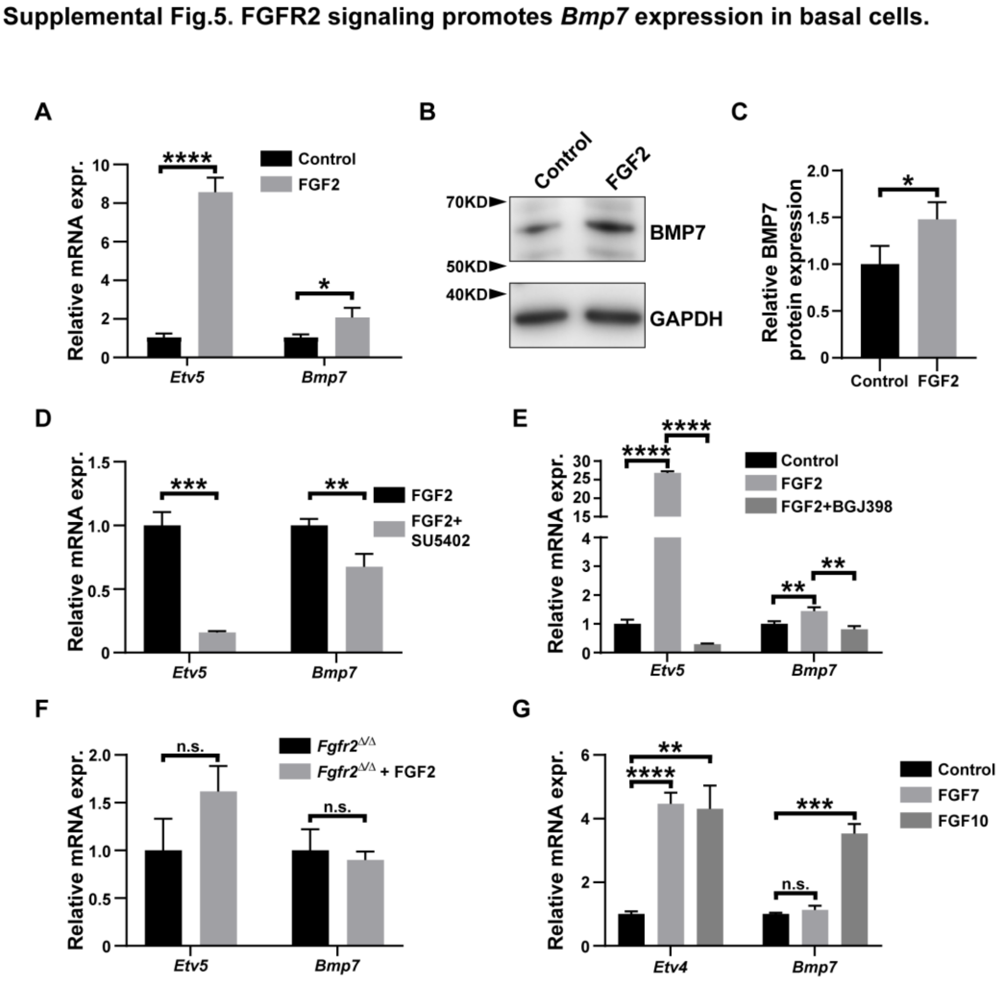

Supplementary Figure S5: FGFR2 signaling promotes Bmp7 expression in basal cells.

(**A**) mRNA expression as detected by qPCR of the FGF signaling target gene *Etv5* and *Bmp7* in wild-type basal cells cultured in basic medium with or without FGF2.

(**B**, **C**) Western blotting (**B**) and quantification (**C**) analysis of BMP7 and the internal control GAPDH protein in the absence or presence of FGF2 stimulation in basal cells. Note that FGF2 significantly upregulated BMP7 protein expression.

(**D**, **E**) mRNA expression as detected by qPCR of the FGF signaling target gene *Etv5* and *Bmp7* cultured in FGF2 basic medium with or without the FGFR inhibitor SU5402 (**D**) or BGJ398 (**E**) in basal cells. Data were mean ± SD (n = 3).

(**F**) mRNA expression as detected by qPCR of the FGF signaling target gene *Etv5* and *Bmp7* in wild-type and $Fgfr2^{\Delta/\Delta}$ basal cells cultured in basic medium with or without FGF2.

(**G**) mRNA expression of *Etv4* and *Bmp7* in basal cells cultured in basic medium with or without FGF7 or FGF10 ligand.

Graph shows mean ± SD. Unpaired Student's t-test was performed for statistical analysis, n.s., not significant, $P \geq 0.05$; * $P < 0.05$; ** $P < 0.01$; *** $P < 0.001$; **** $P < 0.0001$.

Supplemental Fig.6 Basal derived BMP7 promotes luminal expansion.

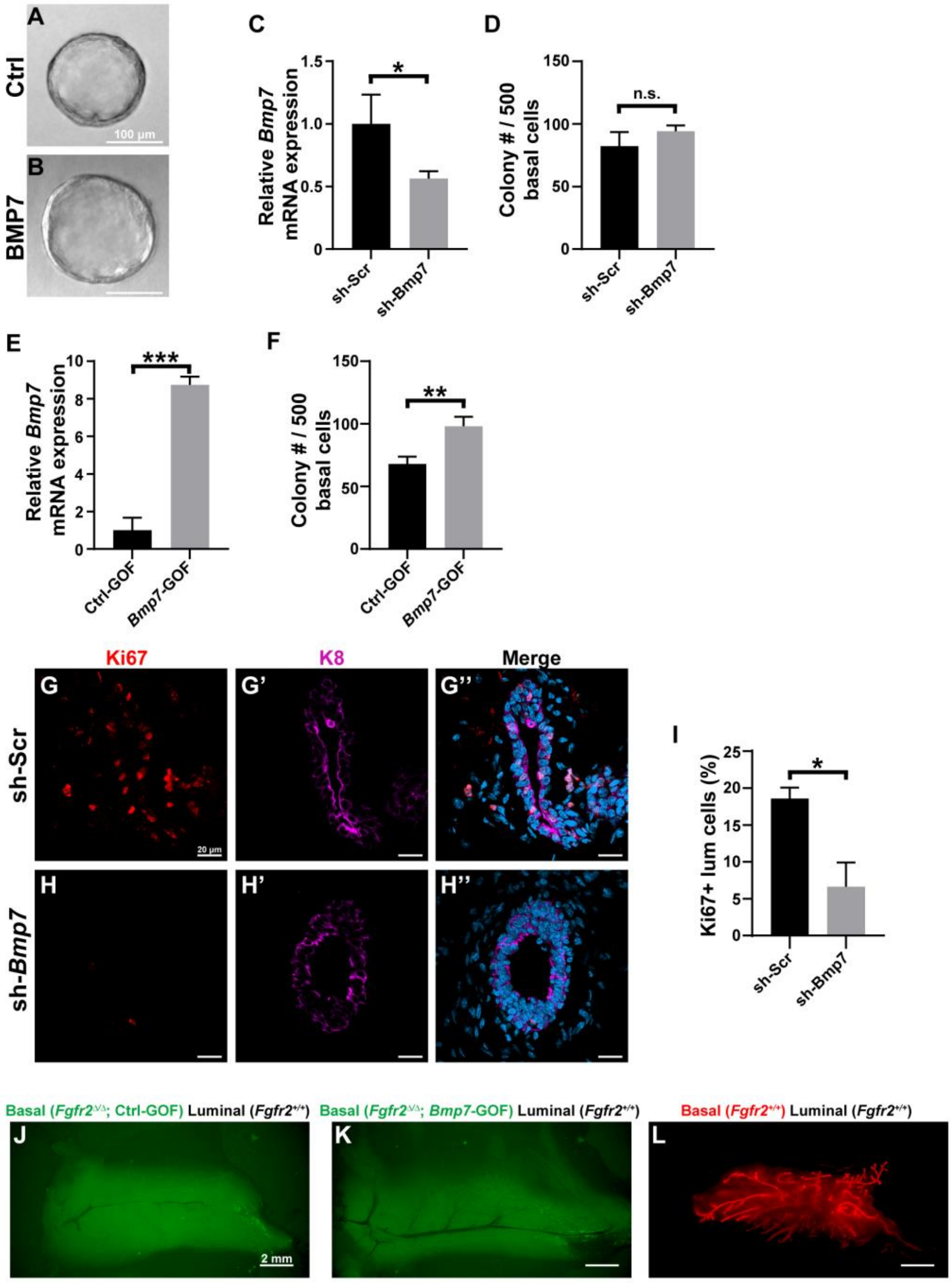

Supplementary Figure S6: Basal derived BMP7 promotes luminal expansion.

(**A**, **B**) Morphology of colonies formed by luminal cells cultured in medium containing with either control PBS (**A**) or 50ng/ml BMP7 (**B**). Scale bars: 100 μm.

(**C**, **D**) Efficiency of *Bmp7* knock-down via shRNA as measured by its relative mRNA expression in basal cells (**C**). Effect of shRNA-based *Bmp7* knock-down on colony formation by 500 basal cells (**D**).

(**E**, **F**) Efficiency of *Bmp7* gain-of-function (GOF) using an overexpression construct based on mRNA expression (**E**, see Methods) and its effect on colony formation of 500 basal cells (**F**).

–(**G**-**I**) Cell proliferation analysis as detected Ki67 immunofluorescence on frozen sections of mammary gland. Transplants were derived from control sh-*Scr* (**G**-**G''**) or sh-*Bmp7* lentivirus (**H**-**H''**) Samples were co-stained with K8 (**G'**, **H'**) and DAPI (**G''**, **H''**) so that only proliferating luminal cells would be quantified (**I**). Scale bars: 20 μm. Data were mean ± SD (more than 500 cells were counted for each sample).

–(**J**-**L**) Whole-mount mammary glands, to which a mixture wild type luminal cells and basal cells of the indicated genotypes were transplanted, observed under a fluorescent stereoscope. (**J**, **K**) *Fgfr2*$^{\Delta/\Delta}$ basal cells were either transfected with a control lentiviral overexpression vector (**J**) or one over-expressing *Bmp7* (**K**). (**L**) A positive transplantation procedure control for (**J**, **K**). Scare bars: 2 mm.

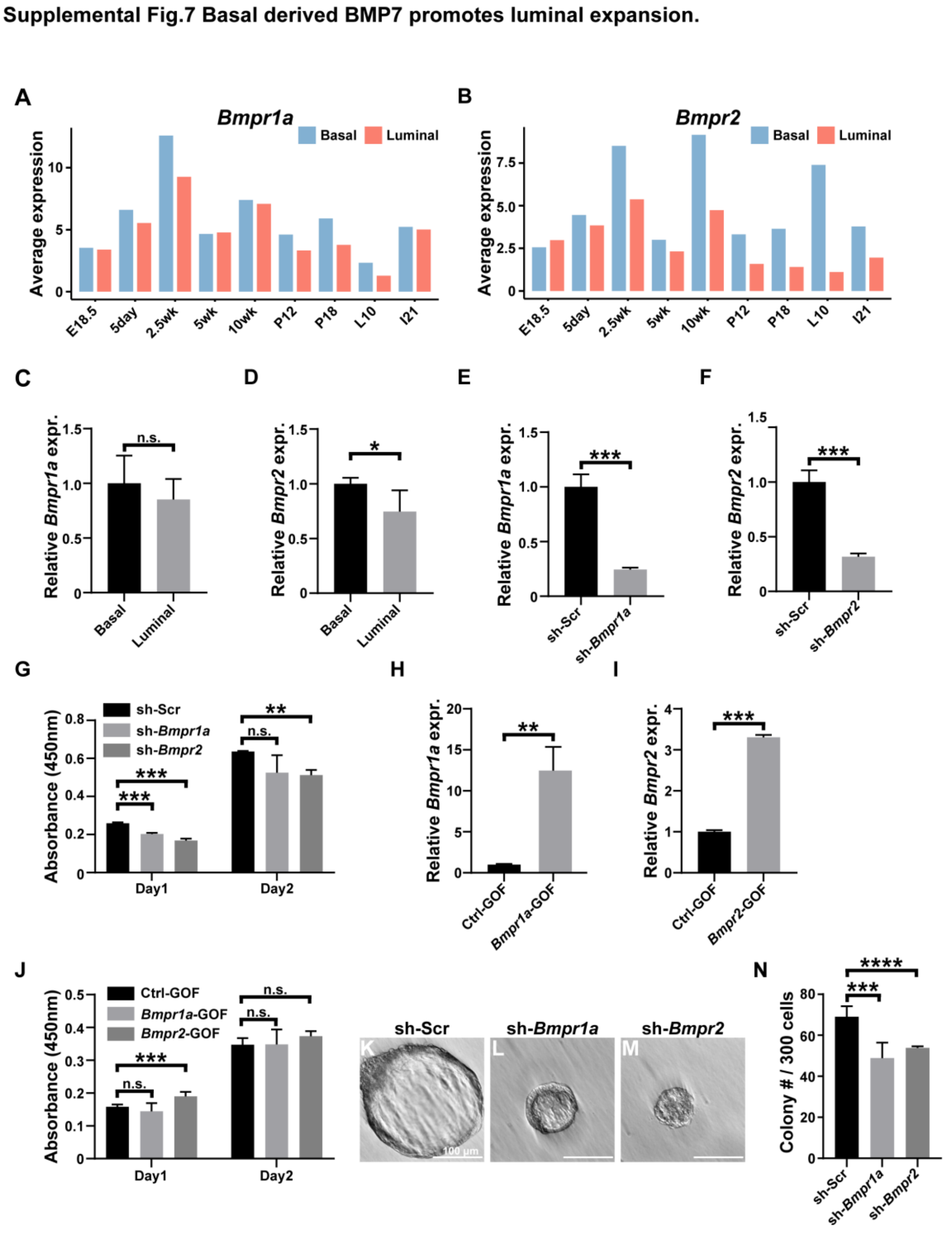

Supplementary Figure S7: Basal derived BMP7 promotes luminal expansion.

(**A, B**) mRNA expression of *Bmpr1a* (**A**) and *Bmpr2* (**B**) in basal and luminal cells at different stages of mouse mammary gland development based on mining scRNA-seq datasets.

(**C, D**) Relative expression of *Bmpr1a* (**C**) and *Bmpr2* (**D**) in sorted luminal and basal cells as measured by qPCR.

(**E**-**G**) Efficiency of *Bmpr1a* (**E**) and *Bmpr2* (**F**) knock-down via shRNA as measured by its relative mRNA expression in luminal cells using qPCR. Effect of shRNA-based *Bmpr* knock-down on HC11 cell proliferation as measured by the CCK8 assay (**G**).

(**H**-**J**) Efficiency of *Bmpr* gain-of-function (GOF) using an expression vector carrying either a control or *Bmpr1a* (**H**) and *Bmpr2* (**I**) cDNA sequence. Their effects on HC11 cell proliferation were measured by the CCK8 assay (**J**). Data were mean ± SD (more than 500 cells were counted for each sample).

(**K**-**N**) Morphology of colonies formed by luminal cells transfected with a lentiviral construct expressing either a control scrambled shRNA (**K**), or the sh-*Bmpr1a* (**L**) and sh-*Bmpr2* (**M**). Colony-forming efficiency was quantified (**N**). n.s. $P \geq 0.05$; *$P<0.05$, ** $P < 0.01$; *** $P < 0.001$; **** $P < 0.0001$.

Supplemental Fig.8 BMP7-BMPR1a/2 signaling mediates basal-to-luminal promotion of epithelial prolifration and expansion.

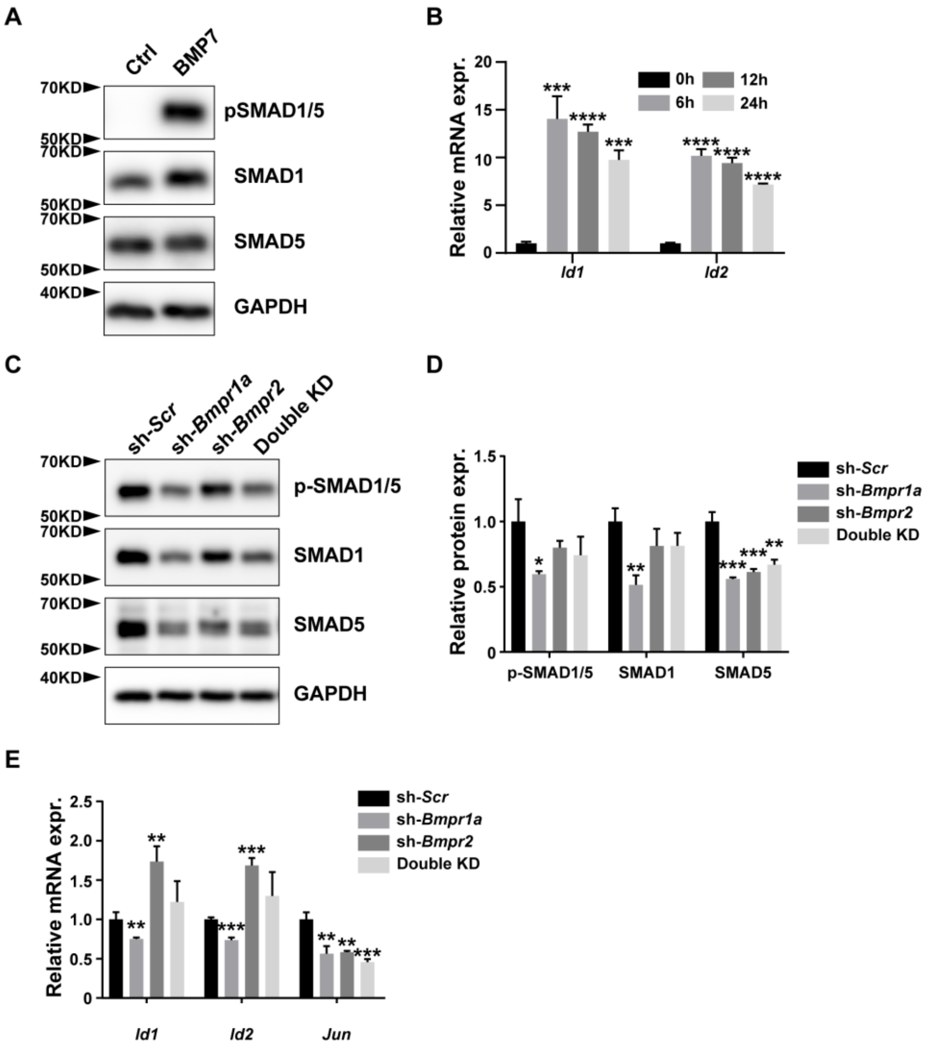

Supplementary Figure S8: BMP7-BMPR1a/2 signaling mediates basal-to-luminal

promotion of epithelial proliferation and expansion in vivo.

(**A**) Western blotting analysis of the indicated BMP signaling components as result of BMP7 stimulation. Note that SMAD1/5, which were un-phosphorylated in their inactive form, became phosphorylated and activated in response to BMP7 stimulation. GAPDH protein was used as an internal control.

(**B**) Relative mRNA expression of the BMP signaling target genes *Id1* and *Id2* by HC11 cells when cultured in medium containing 50ng/ml BMP7 for the durations indicated.

(**C**, **D**) Western blotting analysis of the indicated BMP signaling components by HC11 cell expressing a control shRNA, or shRNAs targeting *Bmpr1a* and *Bmpr2* either alone or in combination (**C**). Levels of SMAD1/5 in both their unphosphorylated (inactive) forms or phosphorylated (activated) forms were quantified (**D**).

(**E**) Relative mRNA expression as measured by qPCR of the BMP signaling target genes *Id1*, *Id2*, and *Jun* by HC11 cell expressing a control shRNA, or shRNAs targeting *Bmpr1a* and *Bmpr2* either alone or in combination.

Data were mean ± SD. n.s. $P \geq 0.05$; *$P<0.05$, ** $P < 0.01$; *** $P < 0.001$; **** $P < 0.0001$.

Supplemental Fig.9 Luminal INHBA is a BMP7 signaling target that promotes expansion of basal stem cell pool.

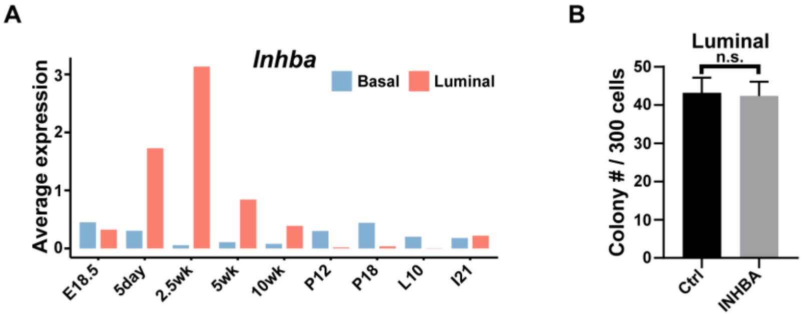

Supplementary Figure S9: Luminal INHBA is a BMP7 signaling target that promotes

expansion of basal stem cell pool.

(**A**) mRNA expression of *Inhba* in the developing mammary gland based on published scRNA-seq datasets.

(**B**) Quantification of colonies formed by 300 luminal cells cultured in medium with or without 5ng/ml INHBA. Data were mean ± SD. n.s. not significant.

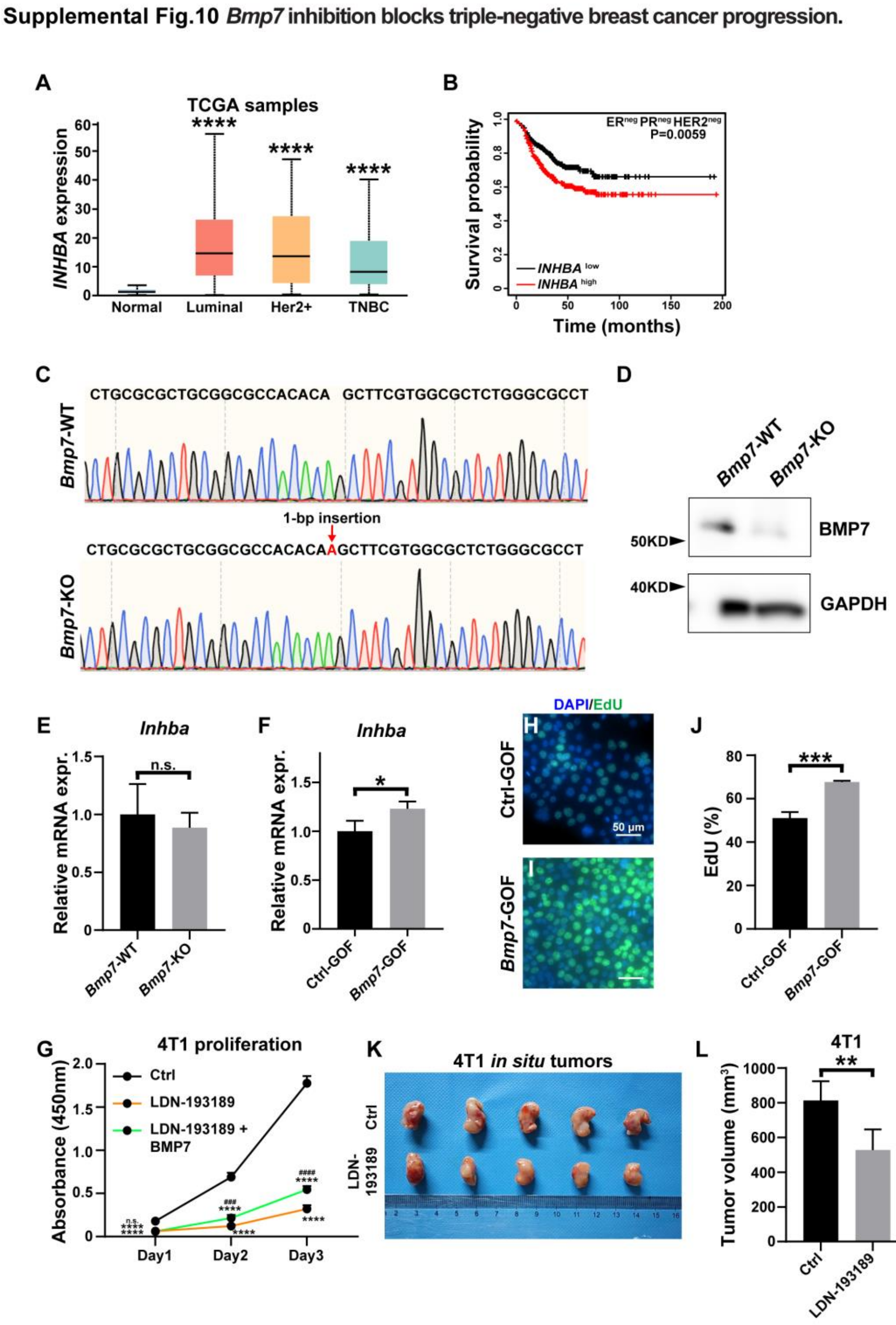

Supplementary Figure S10: BMP7 inhibition blocks triple-negative breast cancer

progression.

(**A**, **B**) mRNA expression in human breast cancer subtypes using the TCGA database (**A**) and Kaplan-Meier survival curves (**B**) of *Inhba*. Survival curves were based on patient data within ~ 150 months post-diagnosis.

(**C**, **D**) Site of *Bmp7* DNA sequence where an additional base was added due to CRISPR-mediate insertion (**C**). (**D**) Protein expression was detected by Western blot to confirmed that *Bmp7* was knocked out.

(**E**, **F**) Relative Inhba mRNA expression as measured by qPCR in *Bmp7*-KO 4T1 cells cultured in medium with serum (**E**) or in *Bmp7*-GOF 4T1 cells cultured in basic medium without serum (**F**).

(**G-I**) Cell proliferation analysis as assessed by EdU-incorporation in 4T1 cells infected with a lentiviral construct over-expressing a control (**G**) or Bmp7 (**H**). (**I**)Data points are the mean ± SD.

(**J**) Effects of 4T1 cell growths as measured by the CCK8 assay in medium without or with the BMP inhibitor LDN-193189 (0.5μM), or with both LDN-193189 and BMP7(50ng/ml). Data points are the mean ± SD.

(**K**, **L**) Tumor growths from 4T1 cells in the cleared fat-pads of nude mice treated with control or LDN193389 (**K**), with tumor volume quantified in (**L**). *P<0.05; *** P < 0.001; **** P < 0.0001. unpaired, two-tailed Student's *t* tests.

SUPPLEMENTARY TABLE1. Primers used in qPCR.

Gene name	Forward sequence (5’ → 3’)	Reverse sequence (5’ → 3’)
Actb	GGCTGTATTCCCCTCCATCG	CCAGTTGGTAACAATGCCATGT
Fgfr2	AATCTCCCAACCAGAAGCGTA	CTCCCCAATAAGCACTGTCCT
Fgfr2b	CCCATCCTCCAAGCTGGACTG	CAGAGCCAGCACTTCTGCATTG
Fgfr2c	AATCTCCCAACCAGAAGCGTA	CTCCCCAATAAGCACTGTCCT
Etv4	CGGAGGATGAAAGGCGGATAC	TCTTGGAAGTGACTGAGGTCC
Etv5	AGGACCCCAGGCTGTACTTT	TGGCCGATTCTTCTGGATAC
Bmp7	ACGGACAGGGCTTCTCCTAC	ATGGTGGTATCGAGGGTGGAA
Bmpr1a	AACAGCGATGAATGTCTTCGAG	GTCTGGAGGCTGGATTATGGG
Bmpr2	TTGGGATAGGTGAGAGTCGAAT	TGTTTCACAAGATTGATGTCCCC
Id1	CCTAGCTGTTCGCTGAAGGC	CTCCGACAGACCAAGTACCAC
Id2	ATGAAAGCCTTCAGTCCGGTG	AGCAGACTCATCGGGTCGT
Id3	CTGTCGGAACGTAGCCTGG	GTGGTTCATGTCGTCCAAGAG
Jun	CAGTCCAGCAATGGGCACATCA	GGAAGCGTGTTCTGGCTATGCA

Inhba	TCACCATCCGTCTATTTCAGCA	CTTCCGAGCATCAACTACTTTCT
Tgfb1	CTCCCGTGGCTTCTAGTGC	GCCTTAGTTTGGACAGGATCTG
Efna1	CTTCACGCCTTTTATCTTGGGC	TGGGGATTATGAGTGATTTTGCC

Total RNA was harvested from mammary glands at the stages indicated. cRNA was prepared as described in Materials and Methods and used as templates for quantitative RT-PCR.
